# ThPOK inhibits the immune escape of gastric cancer cells by inducing STPG1 to inactivate the ERK pathway

**DOI:** 10.1186/s12865-022-00485-5

**Published:** 2022-04-04

**Authors:** Ying Chen, Lili Jiang, Lingli Xia, Gang Zhang, Lan Chen

**Affiliations:** Department of Gastroenterology, The Sixth Hospital of Wuhan, Affiliated Hospital of Jianghan University, 168 Hongkong Road, Jiang’an District, Wuhan, 430015 Hubei China

**Keywords:** ThPOK, STPG1, Immune escape, Gastric cancer

## Abstract

**Background:**

Gastric cancer is the second most frequently diagnosed cancer worldwide. Weak immunogenicity helps cancer cells escape from immune elimination and grow into predominant subpopulations. This study aimed to investigate the effect of Zinc finger and BTB domain containing 7B (Zbtb7b, Alias ThPOK) on T cell activation after coculture with gastric cancer cells.

**Methods:**

Cell Counting Kit-8 assay (CCK-8) was performed to explore the viability of gastric cancer cells. Flow cytometry analysis was used to measure CD3+ T cell proliferation and the ratio of activated IFN-γ+ T cells which were co-incubated with gastric cancer cells (HGC-27, SNU-1). The binding between ThPOK and the promoter of its target sperm tail PG-rich repeat containing 1 (STPG1) was explored using ChIP and luciferase reporter assays. Relative gene expression was quantified using RT-qPCR.

**Results:**

ThPOK was expressed at a low level in gastric cancer tissues and cells at mRNA and protein levels. Gastric cancer patients with lower ThPOK expression had poorer prognosis. ThPOK overexpression suppressed gastric cancer cell viability and increased T cell activation. ThPOK served as a transcription factor for STPG1. STPG1 expression was also at a low level in the tissues and cells of gastric cancer. ThPOK positively regulated the mRNA and protein levels of STPG1 in gastric cancer cells. Moreover, ThPOK was demonstrated to bind with STPG1 promoter. STPG1 upregulation also exerted inhibitory effects on gastric cancer cell viability and T cell activation. Additionally, ThPOK and STPG1 were revealed to inactivate the ERK pathway in gastric cancer cells.

**Conclusion:**

ThPOK inhibits gastric cancer cell viability and increases T cell activation by inducing STPG1 to inactivate the ERK pathway.

**Supplementary Information:**

The online version contains supplementary material available at 10.1186/s12865-022-00485-5.

## Introduction

Gastric cancer (GC) is the fifth most commonly diagnosed malignancy and the third leading cause of cancer related death globally [1]. Helicobacter pylori infection is a primary risk factor for GC, accounting for 90% of new cases of noncardia gastric cancer [2, 3]. There are also other risk factors such as diet, alcohol consumption and smoking [4]. In 2018, over a million new cases are diagnosed of gastric cancer with about 5.7% of all cancer cases worldwide [5]. With a 5-year survival rate of < 25%, gastric cancer patients show no noticeable symptoms in the early stage, and are often observed with distant metastasis that is incurable [6, 7]. Therefore, it is imperative to explore and understand the underlying regulatory mechanisms involved in gastric cancer.

Effector cells preferentially clear high immunogenic tumor cells under the surveillance of the immune system [8]. The weak immunogenicity helps cancer cells to escape from immune elimination and grow into the predominant subpopulations [9]. The effective tumor cell clearance needs both CD4+ and CD8+ T cells. CD4+ T lymphocytes have been reported to regulate the onset and maintenance of the adaptive immune response [10].

Zinc finger and BTB domain containing 7B (Zbtb7b, Alias ThPOK) is a member of the POK family of transcription factors, characterized by a Kruppel-like zinc-finger domain responsible for DNA binding and a regulatory POZ/BTB domain mediating interactions with other factors [11]. ThPOK is a transcription factor that is required by the emergence of memory CD4+ T cells which mediate long term immunity [12]. Increasing studies have revealed the implication of ThPOK in various cancers. For example, ThPOK has been reported to play a critical role in regulating the immune response against colorectal cancer cells [13]. Moreover, it has been documented that ThPOK is selectively expressed in thymocytes while developing to the CD4 lineage. The aberrant expression of ThPOK is associated with high incidence of T-cell lymphomas [14]. However, the function and regulatory mechanism of ThPOK in gastric cancer remain elusive.

Extracellular signal-regulated kinases (ERKs) are part of the mitogen-activated protein kinases (MAPKs) [15], an evolutionary conserved family of proteins modulating mitosis, survival, apoptosis, differentiation, and metabolism [16]. ERKs are activated in response to extracellular stimuli and finely sense changes in the tumor microenvironment, producing adaptive responses of cancer cells [17, 18]. Previous studies revealed that ERK1/2 promotes an immune-evasive phenotype in various tumors including breast [19], liver [20], prostate [21], colon [22], non-small cell lung [23] cancers, and gastrointestinal sarcomas [24]. In the present study, the ERK pathway in gastric cancer cells after coculture with T cells was also studied.

In the present study, we aimed to explore the expression profile and the effect of ThPOK on gastric cancer cell viability and T cell activation after coculture with gastric cancer cells. The findings may provide insights in the exploration of gastric cancer treatments.

## Material and methods

### Bioinformatics analysis

The Kaplan–Meier plotter (https://kmplot.com/analysis/) was used for analyzing the association of ThPOK expression and overall survival of patients with stomach adenocarcinoma based on Pan-cancer Atlas [25]. The target genes of ThPOK were predicted by the HTFtarget database (http://bioinfo.life.hust.edu.cn/hTFtarget#!/).

### Sample collection

The gastric cancer tissue samples (n = 21) and matched adjacent normal tissue samples (n = 21) were collected from gastric cancer patients during operation at the Sixth Hospital of Wuhan, Affiliated Hospital of Jianghan University (Hubei, China). Clinical characteristics of patients were provided in Table 1. All participants had received no radiosurgery or chemosurgery before the operation. The collected tissue samples were immediately frozen in liquid nitrogen at − 80 °C for subsequent experiments. Twenty to thirty micrometer sections (depending on tissue size) were used for RNA/protein extraction. All samples had at least 80% tumor cell content as assessed by two pathologists. Healthy blood donors were healthy physicians from the Sixth Hospital of Wuhan, Affiliated Hospital of Jianghan University. The study was under the approval of the Ethics Committee of the Sixth Hospital of Wuhan, Affiliated Hospital of Jianghan University (Hubei, China). The written informed consents have been signed by all participants before the study.

**Table 1:** Association of ThPOK with clinical features of gastric cancer patients

Parameters	Cases (n)	Relative ThPOK expression		*p* values
		High (n = 10) expression	Low (n = 11)	
*Age (years)*				
≤ 60	8	4	4	0.864
> 60	13	6	7	
*Gender*				
Female	6	3	3	0.89
Male	15	7	8	
*Tumor size*				
≤ 5 cm	18	9	9	0.593
> 5 cm	3	1	2	
*Grade*				
I + II	12	5	7	0.528
III + IV	9	5	4	
*T staging*				
T1 + T2	10	6	4	0.014*
T3 + T4	11	4	7	
*Helicobacter pylori infection*				
Yes	5	1	4	0.157
No	16	9	7	
*Smoking*				
Yes	16	7	9	0.525
No	5	3	2	

ThPOK: zinc finger and BTB domain containing 7B

^*^p value less than 0.05 indicates statistical significance. Chi-square test was performed

### Cell culture

The normal gastric mucosal cells (GES-1, catalog number: CL-0563) were provided by Procell company (Wuhan, Hubei, China). The human gastric cancer cells (HGC-27, catalog number: RCB0500, RCB; SNU-1, CRL-5971, ATCC) were purchased from the Riken Cell bank and American type culture collection. Cells were cultured in Dulbeccoʼs modified Eagleʼs medium (Sigma-Aldrich) containing 10% fetal bovine serum and 1% penicillin/streptomycin (Biochrom, Cambridge, UK) at 37 °C in 5% CO_2_.

### Isolation and identification of T cells

Blood samples were collected from healthy controls. The centrifuge was added with heparin anticoagulant, and the serum was removed. Haemocytes were lysed with red blood cell lysis buffer at room temperature for 10 min. After centrifugation for 5 min, the supernatant was removed. Cell pellets were washed and resuspended in bioclean phosphate buffer saline solution (5 mL) followed by cell counting. CD3 positive cells were selected using flow cytometry.

### In vitro cell co‐culture

10^5^ CFSE (Beijing Solabio Life Sciences Co., Ltd)-labelled CD3^+^ T cells [26] were cocultured with SGC7901 and SNU-1 cells at a ratio of 5:1 in a 96‐well plate with the medium containing 20 IU/mL recombinant human Interleukin‐2 (rhIL‐2), 2 μg/mL anti‐CD3, and 1 μg/mL anti‐CD28. Subsequently, flow cytometry was employed to reveal the percentage of proliferated T cells.

### Cell transfection

The short hairpin RNA against ThPOK (sh-ThPOK#1/2) or STPG1 (sh-STPG1) and corresponding negative control (sh-NC), pcDNA3.1-ThPOK, pcDNA3.1-STPG1 and empty vector (pcDNA3.1-NC) were constructed by RiboBio (Guangzhou, China). The indicated plasmids were transfected into HGC-27 and SNU-1 cells for 48 h using Lipofectamine 3000 (Invitrogen).

### RT-qPCR

The TRIzol Reagent (Sigma-Aldrich) was used for RNA isolation from HGC-27 and SNU-1 cells, and the collected RNA was then reverse transcribed into cDNA by PrimeScript Reverse Transcriptase Kit (Takara, Shiga, Japan). PCR was performed using SYBR ® Premix ExTaq ™ II kit (Takara). The gene expression of ThPOK and STPG1 was calculated with the 2^−∆∆Ct^ method. GAPDH served as the internal control. This experiment was repeated at least three times. The primer sequences are as follows:

ThPOK:

- F: 5′-TCGATTCACCAGGAACGAC-3′,
- R: 5′-TCTTGAGGTCGTAGCTGTG-3′;

STPG1:

- F: 5′-ACTATTACAATGCCTCTGTCTC-3′,
- R: 5′-CGAAAGATCCTCTTTGGGT-3′;

GAPDH:

- F: 5′-CCTCCTGTTCGACAGTCAG-3′,
- R: 5′-CATACGACTGCAAAGACCC-3′;

### Western blotting

Radio Immunoprecipitation Assay (RIPA) lysis buffer was applied for protein extraction from gastric cancer tissues and cells. After culturing on ice for 30 min, gastric tissue samples were centrifuged at 12,000 r/min for 10 min at 4 °C to collect the supernatant. The protein concentration was determined using a BCA kit (Pierce, Rockford, IL, USA). Subsequently, the proteins were isolated by 10% SDS-PAGE. Subsequently, each well was uploaded with 50 μg of protein sample, which was next transferred to PVDF membranes. Non-fat milk (5%) was used to block the membranes for 2 h. The membranes were incubated with the primary antibodies, including anti-ThPOK (#ab228680, 1:500, Abcam), anti-STPG1 (#PA5-49489, 1:2000, Thermo Fisher Scientific, Rochester, NY, USA), anti-p-ERK-1/2 (#44-680G, 1:1000, Abcam), anti-ERK-1/2 (#ab184699, 1:10,000, Abcam) at 4 °C overnight with GAPDH as the internal reference. Next, these membranes were incubated with the secondary antibody for 1 h in the dark at room temperature. The protein bands were visualized using the enhanced chemiluminescence (ECL) detection system (Thermo Fisher Scientific).

### Cell counting kit-8 assay

The transfected HGC-27 and SNU-1 cells (1000 cells/well) were grown into 96-well plates. At specific time point (0, 24, 48, 72 h), 10 μL of CCK-8 solution (Dojindo, Tokyo, Japan) was added into each well and cultured for another 4 h. Microplate reader (Reagen, Shenzhen, China) was used to detect the absorbance of HGC-27 and SNU-1 cells at 450 nm.

### Flow cytometry analysis

After preparing the single cell suspension and resuspending the cells in the staining buff. PerCP-CD3 and Pacific blue-interferon-γ were added to culture with T cells. After immobilization and permeabilization, Pacific blue-IFN-γ was used. BD fluorescent-activated cell sorting canto II was used to measure the cells and flow Jo software was used for analysis [27, 28].

### Luciferase reporter assay

Luciferase reporter assay was performed to explore the interaction between ThPOK and STPG1 promoter. Sequence of STPG1 promoter was subcloned into PGL3 vector (Promega), and the vectors carrying STPG1 promoter were cotransfected with sh-ThPOK#1/2 or sh-NC with Lipofectamine 3000 into HGC-27 and SNU-1 cells for 48 h. Next, the Dual-Luciferase Reporter Assay System (Promega) was used to analyze the relative luciferase activity of STPG1 promoter in gastric cancer cells.

### Chromatin immunoprecipitation (ChIP) assay

ChIP assay kits (Upstate Biotechnology, Temacula, CA) were used to perform ChIP assays. The HGC-27 and SNU-1 cells were cross-linked with 1% formaldehyde (Thermo Fisher) for 10 min. After being centrifuged at 12,000*g* for 10 min at 4 °C, the obtained cell lysate was incubated with anti-ThPOK or anti-IgG. IgG functioned as a negative control. The collected DNA from the complexes by the DNA extraction kits (QIAGEN) was subjected to RT-qPCR analysis.

### Statistical analysis

The statistical analysis was performed with SPSS 21.0 software (IBM, Armonk, NY, USA). The results of experiments that were independently conducted for 3 times are shown by the mean ± SD. The Student’s t-test evaluated the two-group differences, while difference among multiple groups was compared by ANOVA. The Kaplan–Meier method was used to detect the survival in gastric cancer patients. *p* < 0.05 was regarded as statistical significance.

## Results

### ThPOK is downregulated in the tissues and cells of gastric cancer

**Fig. 1:**
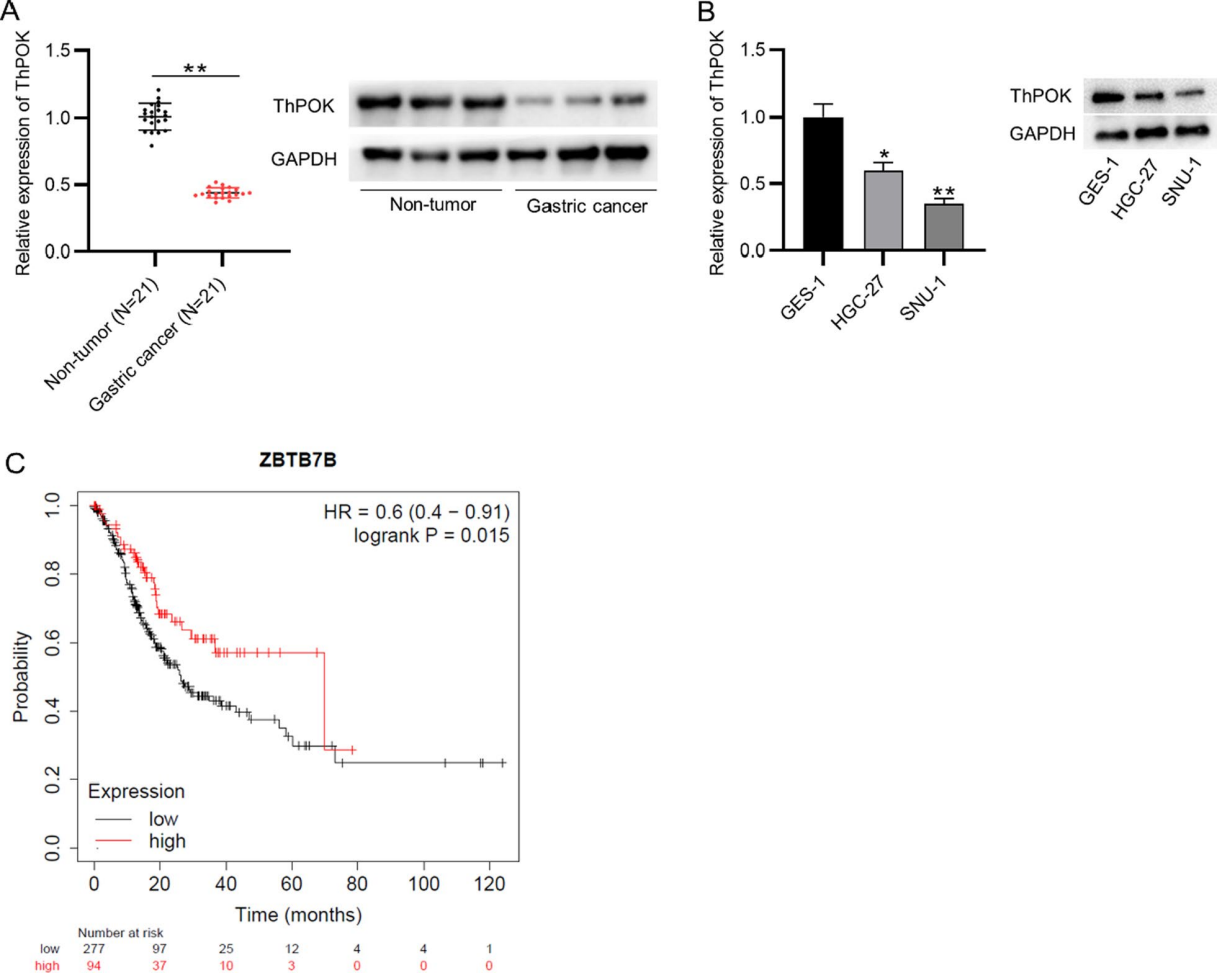
ThPOK is downregulated in the tissues and cells of gastric cancer. **A** ThPOK mRNA and protein expression in the tissues of gastric cancer patients and adjacent normal tissues was assessed using RT-qPCR analysis and western blotting. **B** The mRNA and protein levels of ThPOK in gastric cancer cells (HGC-27, SNU-1) and normal gastric mucosal cells (GES-1) were assessed by RT-qPCR and western blotting. **C** Kaplan–Meier Plotter (https://kmplot.com/analysis/) was used to predict the prognosis in gastric cancer patients with high or low level of ThPOK. *p < 0.05, **p < 0.01

ThPOK expression profile in the gastric cancer tissue samples was explored using RT-qPCR analysis. The downregulation of ThPOK was confirmed in gastric tissues compared with the normal adjacent tissues. The low protein expression of ThPOK was also confirmed in the tissues of gastric cancer patients (Fig. 1a). ThPOK expression is significantly associated with the T Staging of gastric cancer patients (Table 1). We measured the ThPOK expression in gastric cancer cells and normal gastric mucosal cells, the result of RT-qPCR and western blotting analyses indicated that mRNA and protein levels of ThPOK were both reduced in cell lines of gastric cancer than the control (Fig. 1b). The prediction on the Kaplan–Meier Plotter showed that gastric cancer patients with high levels of ThPOK were predicted with better prognosis (Fig. 1c).

### ThPOK inhibits the immune escape of gastric cancer cells

**Fig. 2:**
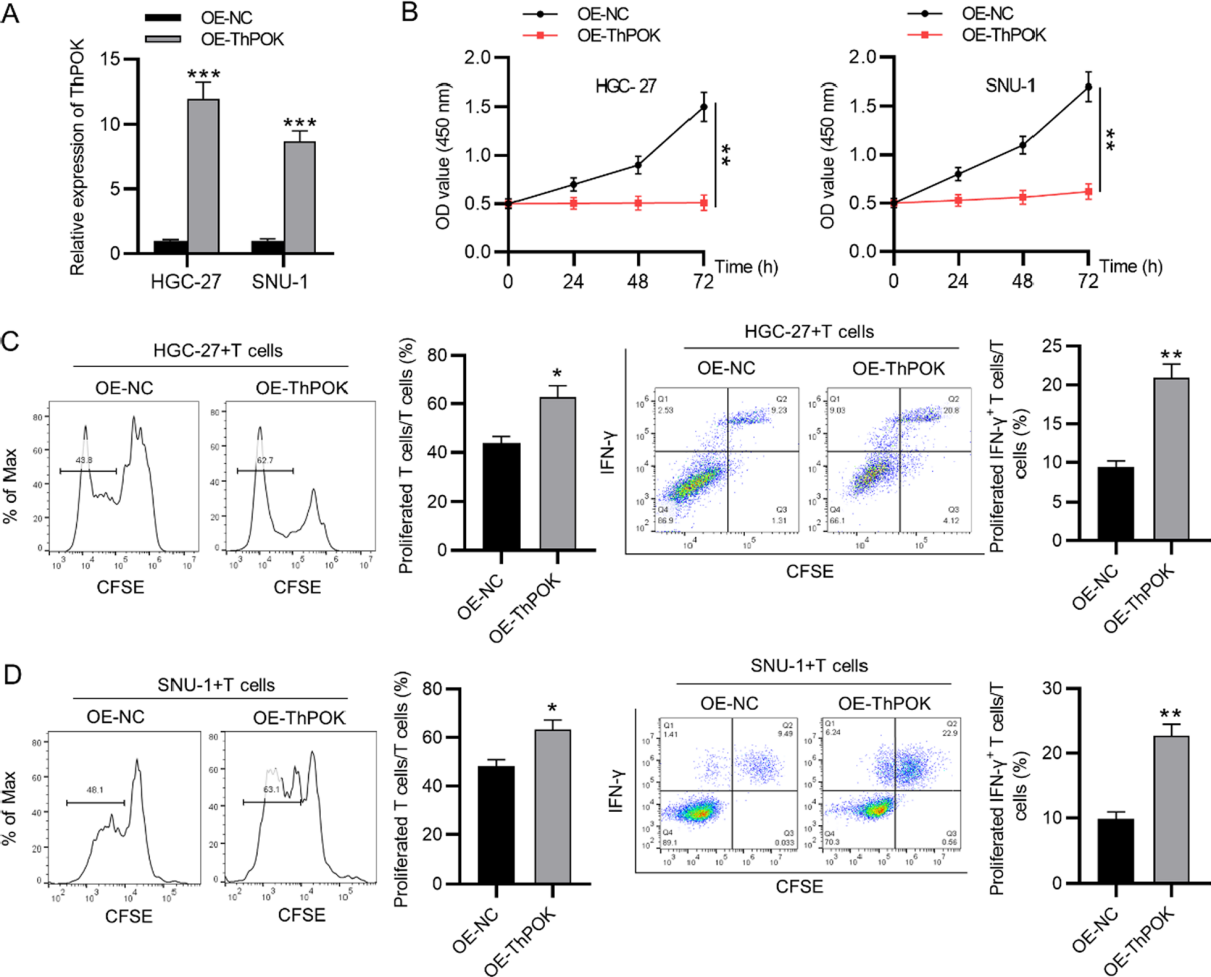
ThPOK inhibits the immune escape of gastric cancer cells. **A** ThPOK expression in gastric cancer cells transfected with OE-ThPOK and OE-NC was measured by RT-qPCR. **B** Gastric cancer cell viability was determined by CCK-8 assays after ThPOK overexpression. **C** and **D** Flow cytometry analysis was used to detect T cell proliferation and proportion of IFN-γ+ T cells after coculture with gastric cancer cells (HGC-27, SNU-1). *p < 0.05, **p < 0.01, ***p < 0.001

Gain-of-function assays were performed to investigate the effect of ThPOK on T cellactivation after coculture with gastric cancers. First, the overexpression efficiency of ThPOK in gastric cancer cells was confirmed by RT-qPCR (Fig. 2a). According to the result of CCK-8 assay, the viability of gastric cancer cells was reduced after ThPOK overexpression (Fig. 2b). Gastric cancer cells (HGC-27, SNU-1) were co-cultured with T cells in vitro. The result of flow cytometry analysis indicated that the proliferation ability of CD3+ T cells as well as the ratio of IFN-γ+ T cells were both increased after ThPOK upregulation in comparison with the control group (Fig. 2c, d).

### ThPOK induces upregulation of STPG1 in gastric cancer cells at the transcription level

**Fig. 3:**
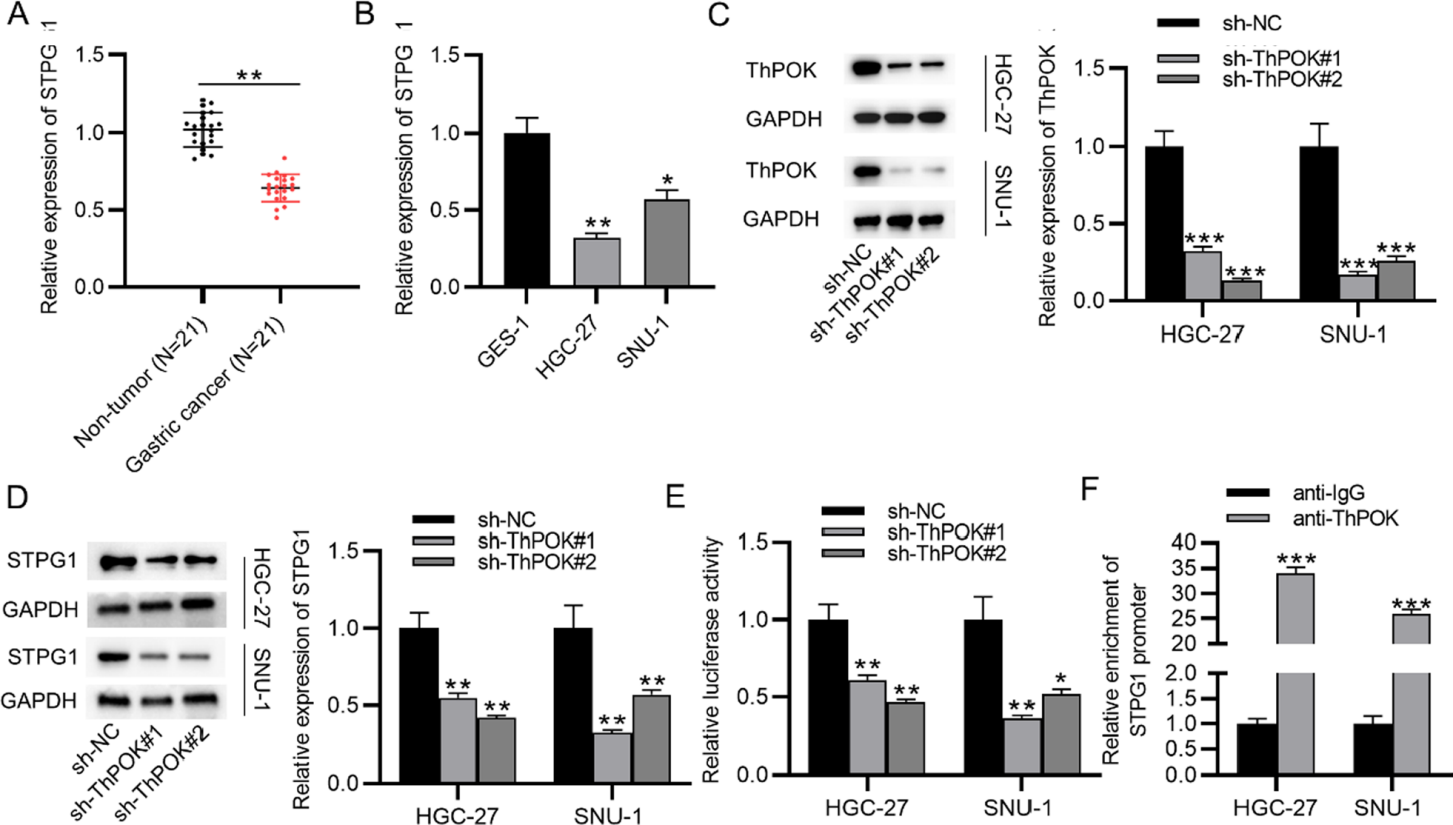
ThPOK induces upregulation of STPG1 in gastric cancer cells at the transcription level. **A** STPG1 level in gastric cancer tissues (n = 21) and adjacent normal tissues (n = 21) was assessed by RT-qPCR. **B** The expression of STPG1 in gastric cancer cells (HGC-27, SNU-1) and normal gastric mucosal cells (GES-1) was assessed by RT-qPCR. **C** RT-qPCR and western blotting were used to measure the knockdown efficiency of ThPOK in gastric cancer cells. **D** STPG1 mRNA and protein expression in gastric cancer cells posttransfection of sh-ThPOK#1/2. **E** The binding between ThPOK and the promoter region of STPG1 after the transfection of sh-ThPOK#1/2 was explored by luciferase reporter assay. **F** ChIP assay was used to verify the binding between ThPOK and STPG1 promoter. *p < 0.05, **p < 0.01, ***p < 0.001

STPG1 was on the top of the list of ThPOK target genes (Additional file 1) and was selected for further research. The result of RT-qPCR analysis indicated that STPG1 expression was lower in the tissues of gastric cancer patients than adjacent normal tissues (Fig. 3a). We measured its expression in gastric cancer cells using RT-qPCR. The result demonstrated that the low level of STPG1 was confirmed in cells of gastric cancer compared with the normal gastric mucosal cells (GES-1) (Fig. 3b). The knockdown efficiency of ThPOK in gastric cancer cells was verified. After the transfection of sh-ThPOK#1/2, the ThPOK mRNA and protein levels were reduced in gastric cancer cells (Fig. 3c). The effect of ThPOK knockdown on the expression of STPG1 was further explored. Moreover, a reduction in the mRNA and protein levels of STPG1 was observed in gastric cancer cells after the transfection of sh-ThPOK#1/2. Thus, ThPOK positively regulated STPG1 expression in gastric cancer cells (Fig. 3d). The interaction between ThPOK and promoter region of STPG1 was assessed using luciferase reporter assay. After the transfection of sh-ThPOK#1/2, the luciferase activity of the STPG1 promoter was reduced in gastric cancer cells (Fig. 3e). The result of ChIP assay showed that STPG1 was enriched in the precipitates of anti-ThPOK antibodies, which further confirmed the binding between ThPOK and STPG1 promoter (Fig. 3f).

### STPG1 inhibits the immune escape of gastric cancer cells

**Fig. 4:**
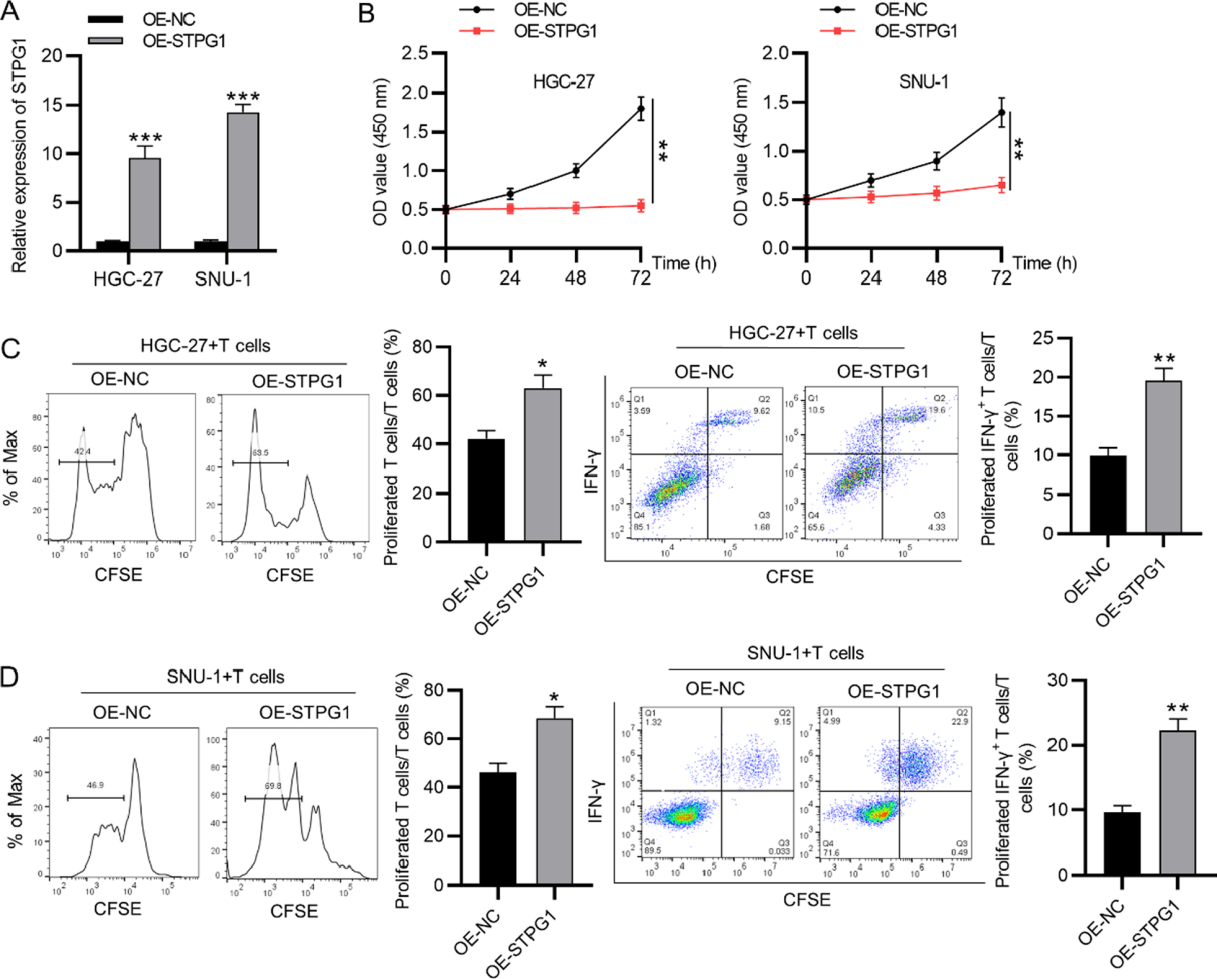
STPG1 inhibits the immune escape of gastric cancer cells. **A** STPG1 expression in gastric cancer cells posttransfection of OE-STPG1 or OE-NC was detected by RT-qPCR. **B** CCK-8 assay was conducted to assess the viability of gastric cancer cells with overexpression of STPG1. **C** and **D** Flow cytometry analysis was used to detect the percentage of proliferated CD3+ T cells and the proportion of IFN-γ+ T cells after co-culture with gastric cancer cells (HGC-27, SNU-1) which were transfected with STPG1 overexpression vectors. *p < 0.05, **p < 0.01, ***p < 0.001

We further explored whether STPG1 affected T cell activation. The overexpression efficiency of STPG1 in gastric cancer cells was confirmed by RT-qPCR (Fig. 4a). The result of CCK-8 assay indicated that overexpressed STPG1 suppressed the viability of gastric cancer cells (Fig. 4b). According to flow cytometry analysis, STPG1 overexpression promoted the proliferation ability of CD3+ T cells as well as the ratio of IFN-γ+ T cells (Fig. 4c, d).

### ThPOK and STPG1 inactivate the ERK pathway

**Fig. 5:**
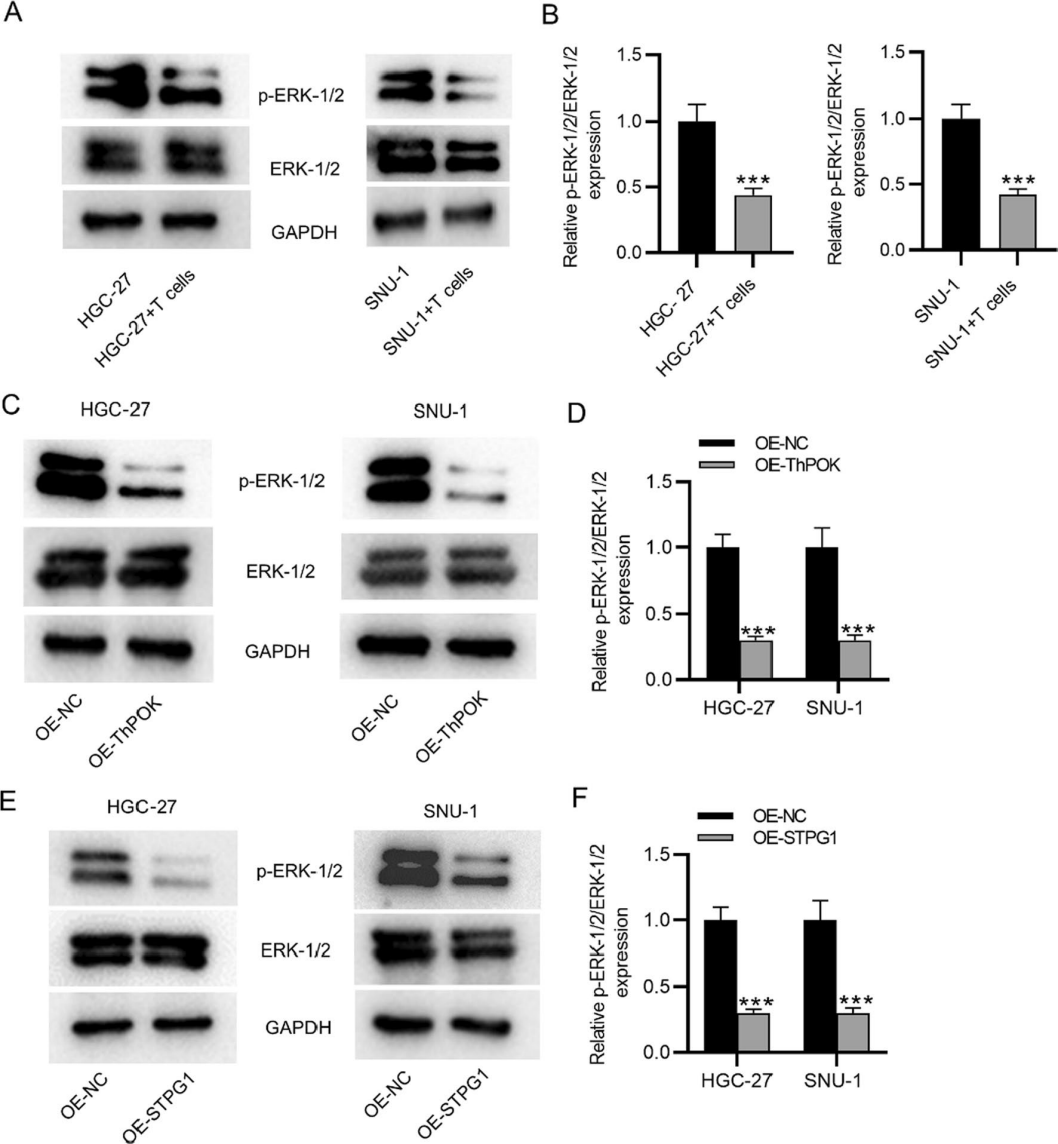
ThPOK and STPG1 inactivate the ERK pathway. **A** and **B** p-ERK-1/2 and ERK-1/2 levels in gastric cancer cells after coculture with T cells. **C** and **D** Western blotting was used to detect the protein levels of p-ERK-1/2 and ERK-1/2 in gastric cancer cells transfected with OE-ThPOK or OE-NC. **E** and **F** The protein levels of p-ERK-1/2 and ERK-1/2 in gastric cancer cells after the transfection of OE-STPG1 or OE-NC. ***p < 0.001

We further explored the effect of ThPOK and STPG1 on the downstream ERK signaling pathways. Figure 5a and b revealed that after coculture with T cells, the p-ERK-1/2 levels in gastric cancers were decreased. The result of western blotting indicated that the protein level of p-ERK-1/2 was significantly decreased after the overexpression of ThPOK and STPG1 in gastric cancer cells, and the ratio of p-ERK-1/2 to ERK-1/2 also showed a significant decrease in gastric cancer cells (Fig. 5c–f). All original western blot images were provided in Additional file 2.

### ThPOK promotes T cell activation by STPG1

**Fig. 6:**
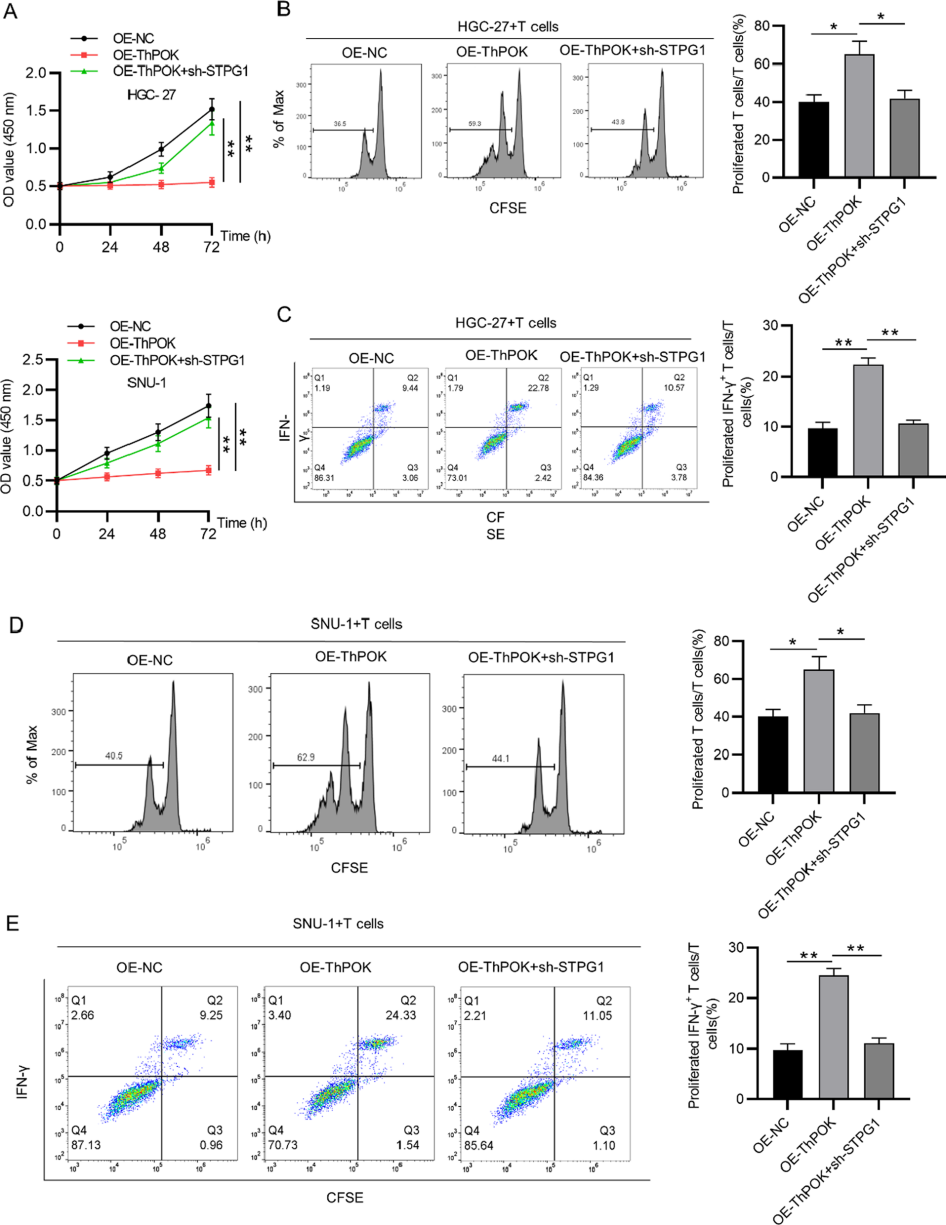
ThPOK promotes T cell activation by STPG1. **A** Viability of gastric cancer cells under the conditions of OE-NC, OE-ThPOK, and OE-ThPOK + sh-STPG1 was detected by CCK-8 assay. Flow cytometry analysis was used to detect **B** and **D** the percentage of proliferated CD3+ T cells and **C** and **E** the proportion of IFN-γ+ T cells after coculture with gastric cancer cells (HGC-27, SNU-1) under the conditions of OE-NC, OE-ThPOK, and OE-ThPOK + sh-STPG1. *p < 0.05, **p < 0.01

Gastric cancer cells (HGC-27, SNU-1) were co-cultured with T cells in vitro. The viability of gastric cancer cells and the proliferation ability of CD3+ T cells as well as the ratio of IFN-γ+ T cells in response to ThPOK overexpression and STPG1 knockdown were evaluated using CCK-8 assays and flow cytometry analyses, respectively. Silencing of STPG1 rescued the suppressive effect of ThPOK overexpression on the viability of gastric cancer cells (Fig. 6a). The inhibitory impacts of ThPOK overexpression on the percentage of proliferated CD3+ T cells as well as the ratio of IFN-γ+ T cells were also partially restored by STPG1 knockdown (Fig. 6b–e).

## Discussion

In our study, ThPOK was found to function as a tumor suppressor in gastric cancer. The expression of ThPOK was significantly downregulated in gastric cancer tissues and cells at both mRNA and protein levels. Moreover, the lower level of ThPOK was associated with poorer prognosis in gastric cancer patients. Gain-of-function assays showed that ThPOK overexpression suppressed the viability of gastric cancer cell lines and promoted T cell activation after coculture with gastric cancer cells.

ThPOK is an important transcription regulator and is critically involved in the commitment of some leucocytic lineages including helper, cytotoxic and natural killer T cells that define the aggressiveness and prognosis of various cancers, gastric cancer included [29]. In our study, ThPOK was revealed to serve as the transcription factor for STPG1. The low expression of STPG1 was also confirmed in the tissues and cells of gastric cancer. The mRNA and protein expression of STPG1 was positively modulated by ThPOK in gastric cancer cells. Moreover, the result of luciferase reporter assay and ChIP assay indicated that ThPOK bound with the promoter of STPG1. The function of STPG1 on gastric cancer cell behaviors was also explored. The CCK-8 assay showed the decreased gastric cancer cell viability after the transfection of STPG1 overexpression vectors. Moreover, STPG1 overexpression was also found to enhance T cell activation after coculture with gastric cancer cells. The role of STPG1 in cancer has been rarely investigated. A previous study has built a ten-gene signature including STPG1, and identified that this signature is correlated with the survival and immune infiltrating in uveal melanoma [30]. Our study further demonstrated the function of STPG1 on T cell activation after coculture with gastric cancer cells.

The ERK signaling pathway is critically involved in cancer progression. The proteins extracellular signal-regulated kinase 1 (ERK1) and ERK2 are reported to control the cellular processes by widely phosphorylating diverse substrates, and their dysregulation leads to various diseases [31]. In the present study, we found that the protein levels of phosphorylated ERK-1/2 and the ratio of p-ERK-1/2/ERK-1/2 were both significantly decreased after ThPOK overexpression or STPG1 upregulation in gastric cancer cells, which indicated that ThPOK and STPG1 exerted inhibitory effects on the ERK signaling pathway. The ERK signaling pathway has also been reported to be implicated in multiple malignancies including prostate cancer [32], ovarian cancer [33], lung cancer [34]. For example, miR-302b inhibits conversion of the G1/S by negatively regulating CDK2 to inactivate the ERK signaling pathway in gastric cancer [35]. Since the aberrant activation of signal transducing proteins is closely associated with cancer progression, the exploration for the inhibitors of ERK pathway is of vital importance [36]. Our study has demonstrated that both ThPOK and STPG1 inactivated the ERK signaling in gastric cancer cells. ERKs are reported to be promising therapeutic targets for investigation of anticancer drugs.

## Conclusions

In conclusion, ThPOK was downregulated in gastric cancer tissues and cells and its low expression predicted poor prognosis in gastric cancer patients. ThPOK inhibited gastric cancer cell viability and promoted T cell activation by transcriptionally inducing STPG1 to inactivate the ERK signaling. The findings in our study may provide new clues for the treatment of gastric cancer.

## Supplementary Information

**Additional file 1**. Target genes of transcriptional factor ThPOK.

**Additional file 2**. Original western blot images.

## Data Availability

All data from this study are available in this published article.

## Abbreviations

STPG1: Sperm tail PG-rich repeat containing 1
GC: Gastric cancer
Zbtb7b: Zinc finger and BTB domain containing 7B
