# Supplementary material for: ThPOK inhibits the immune escape of gastric cancer cells by inducing STPG1 to inactivate the ERK pathway
Source: BMC Immunol. 2022 Apr 4;23:16. doi: 10.1186/s12865-022-00485-5 (PMC8981657; doi:10.1186/s12865-022-00485-5)
Supplement: Supplementary file 1 — Additional file 1. Target genes of transcriptional factor ThPOK. [file 12865_2022_485_MOESM1_ESM.docx]

**Additional file Target genes of transcriptional factor ThPOK**

| **TF_name** | **target_id** | **target_name** | **target_synonyms** |
| --- | --- | --- | --- |
| ZBTB7B | ENSG00000001460 | STPG1 | C1orf201, MAPO2 |
| ZBTB7B | ENSG00000002330 | BAD | BBC2, BCL2L8 |
| ZBTB7B | ENSG00000002834 | LASP1 | Lasp-1, MLN50 |
| ZBTB7B | ENSG00000004838 | ZMYND10 | BLU, CILD22, FLU |
| ZBTB7B | ENSG00000005001 | PRSS22 | BSSP-4, SP001LA, hBSSP-4 |
| ZBTB7B | ENSG00000005238 | FAM214B | KIAA1539 |
| ZBTB7B | ENSG00000005243 | COPZ2 | zeta2-COP |
| ZBTB7B | ENSG00000005448 | WDR54 | - |
| ZBTB7B | ENSG00000005486 | RHBDD2 | NPD007, RHBDL7 |
| ZBTB7B | ENSG00000005844 | ITGAL | CD11A, LFA-1, LFA1A |
| ZBTB7B | ENSG00000006007 | GDE1 | 363E6.2, MIR16 |
| ZBTB7B | ENSG00000006025 | OSBPL7 | ORP7 |
| ZBTB7B | ENSG00000006062 | MAP3K14 | FTDCR1B, HS, HSNIK, NIK |
| ZBTB7B | ENSG00000006327 | TNFRSF12A | CD266, FN14, TWEAKR |
| ZBTB7B | ENSG00000006712 | PAF1 | F23149_1, PD2 |
| ZBTB7B | ENSG00000007384 | RHBDF1 | C16orf8, Dist1, EGFR-RS, gene-89, gene-90, hDist1 |
| ZBTB7B | ENSG00000008130 | NADK | dJ283E3.1 |
| ZBTB7B | ENSG00000008517 | IL32 | IL-32alpha, IL-32beta, IL-32delta, IL-32gamma, NK4, TAIF, TAIFa, TAIFb, TAIFc, TAIFd |
| ZBTB7B | ENSG00000008853 | RHOBTB2 | DBC2 |
| ZBTB7B | ENSG00000009780 | FAM76A | - |
| ZBTB7B | ENSG00000009830 | POMT2 | LGMD2N, MDDGA2, MDDGB2, MDDGC2 |
| ZBTB7B | ENSG00000010219 | DYRK4 | - |
| ZBTB7B | ENSG00000010292 | NCAPD2 | CAP-D2, CNAP1, hCAP-D2 |
| ZBTB7B | ENSG00000010295 | IFFO1 | HOM-TES-103, IFFO |
| ZBTB7B | ENSG00000010361 | FUZ | FY, NTD |
| ZBTB7B | ENSG00000010626 | LRRC23 | LRPB7 |
| ZBTB7B | ENSG00000011485 | PPP5C | PP5, PPP5, PPT |
| ZBTB7B | ENSG00000012061 | ERCC1 | COFS4, RAD10, UV20 |
| ZBTB7B | ENSG00000012171 | SEMA3B | hsa-mir-6872 |
| ZBTB7B | ENSG00000013503 | POLR3B | C128, HLD8, INMAP, RPC2 |
| ZBTB7B | ENSG00000013523 | ANGEL1 | Ccr4e, KIAA0759 |
| ZBTB7B | ENSG00000017797 | RALBP1 | RIP1, RLIP1, RLIP76 |
| ZBTB7B | ENSG00000022567 | SLC45A4 | - |
| ZBTB7B | ENSG00000025293 | PHF20 | C20orf104, GLEA2, HCA58, NZF, TDRD20A, TZP |
| ZBTB7B | ENSG00000025770 | NCAPH2 | CAPH2 |
| ZBTB7B | ENSG00000034533 | ASTE1 | HT001 |
| ZBTB7B | ENSG00000034713 | GABARAPL2 | ATG8, ATG8C, GATE-16, GATE16, GEF-2, GEF2 |
| ZBTB7B | ENSG00000036549 | ZZZ3 | ATAC1 |
| ZBTB7B | ENSG00000036672 | USP2 | UBP41, USP9 |
| ZBTB7B | ENSG00000037897 | METTL1 | C12orf1, TRM8, TRMT8, YDL201w |
| ZBTB7B | ENSG00000039650 | PNKP | AOA4, EIEE10, MCSZ, PNK |
| ZBTB7B | ENSG00000041880 | PARP3 | ADPRT3, ADPRTL2, ADPRTL3, ARTD3, IRT1, PADPRT-3 |
| ZBTB7B | ENSG00000042493 | CAPG | AFCP, HEL-S-66, MCP |
| ZBTB7B | ENSG00000043143 | JADE2 | JADE-2, PHF15 |
| ZBTB7B | ENSG00000047578 | KIAA0556 | JBTS26 |
| ZBTB7B | ENSG00000049541 | RFC2 | RFC40 |
| ZBTB7B | ENSG00000050438 | SLC4A8 | NBC3, NDCBE |
| ZBTB7B | ENSG00000050820 | BCAR1 | CAS, CAS1, CASS1, CRKAS, P130Cas |
| ZBTB7B | ENSG00000052749 | RRP12 | KIAA0690 |
| ZBTB7B | ENSG00000054148 | PHPT1 | CGI-202, HEL-S-132P, HSPC141, PHP, PHP14 |
| ZBTB7B | ENSG00000061936 | SFSWAP | SFRS8, SWAP |
| ZBTB7B | ENSG00000063322 | MED29 | IXL, MED2 |
| ZBTB7B | ENSG00000064012 | CASP8 | ALPS2B, CAP4, Casp-8, FLICE, MACH, MCH5 |
| ZBTB7B | ENSG00000064547 | LPAR2 | EDG-4, EDG4, LPA-2, LPA2 |
| ZBTB7B | ENSG00000064961 | HMG20B | BRAF25, BRAF35, HMGX2, HMGXB2, PP7706, SMARCE1r, SOXL, pp8857 |
| ZBTB7B | ENSG00000065000 | AP3D1 | ADTD, HPS10, hBLVR |
| ZBTB7B | ENSG00000065057 | NTHL1 | FAP3, NTH1, OCTS3, hNTH1 |
| ZBTB7B | ENSG00000065970 | FOXJ2 | FHX |
| ZBTB7B | ENSG00000067182 | TNFRSF1A | CD120a, FPF, TBP1, TNF-R, TNF-R-I, TNF-R55, TNFAR, TNFR1, TNFR55, TNFR60, p55, p55-R, p60 |
| ZBTB7B | ENSG00000067369 | TP53BP1 | 53BP1, TDRD30, TP53, p202, p53BP1 |
| ZBTB7B | ENSG00000068001 | HYAL2 | LUCA2 |
| ZBTB7B | ENSG00000068028 | RASSF1 | 123F2, NORE2A, RASSF1A, RDA32, REH3P21 |
| ZBTB7B | ENSG00000068724 | TTC7A | GIDID, MINAT, TTC7 |
| ZBTB7B | ENSG00000068745 | IP6K2 | IHPK2, PIUS |
| ZBTB7B | ENSG00000068976 | PYGM | - |
| ZBTB7B | ENSG00000070404 | FSTL3 | FLRG, FSRP |
| ZBTB7B | ENSG00000070444 | MNT | MAD6, MXD6, ROX, bHLHd3 |
| ZBTB7B | ENSG00000071462 | WBSCR22 | HASJ4442, HUSSY-3, MERM1, PP3381, WBMT |
| ZBTB7B | ENSG00000072682 | P4HA2 | - |
| ZBTB7B | ENSG00000072778 | ACADVL | ACAD6, LCACD, VLCAD |
| ZBTB7B | ENSG00000072818 | ACAP1 | CENTB1 |
| ZBTB7B | ENSG00000072958 | AP1M1 | AP47, CLAPM2, CLTNM, MU-1A |
| ZBTB7B | ENSG00000073067 | CYP2W1 | - |
| ZBTB7B | ENSG00000076108 | BAZ2A | TIP5, WALp3 |
| ZBTB7B | ENSG00000077235 | GTF3C1 | TFIIIC, TFIIIC220, TFIIICalpha |
| ZBTB7B | ENSG00000077935 | SMC1B | SMC1BETA, SMC1L2 |
| ZBTB7B | ENSG00000078246 | TULP3 | TUBL3 |
| ZBTB7B | ENSG00000078668 | VDAC3 | HD-VDAC3, VDAC-3 |
| ZBTB7B | ENSG00000079387 | SENP1 | SuPr-2 |
| ZBTB7B | ENSG00000080371 | RAB21 | - |
| ZBTB7B | ENSG00000080603 | SRCAP | - |
| ZBTB7B | ENSG00000081248 | CACNA1S | CACNL1A3, CCHL1A3, Cav1.1, HOKPP, HOKPP1, MHS5, TTPP1, hypoPP |
| ZBTB7B | ENSG00000081791 | KIAA0141 | DELE |
| ZBTB7B | ENSG00000082458 | DLG3 | MRX, MRX90, NEDLG, PPP1R82, SAP102, XLMR |
| ZBTB7B | ENSG00000083812 | ZNF324 | ZF5128, ZNF324A |
| ZBTB7B | ENSG00000083838 | ZNF446 | ZKSCAN20, ZSCAN30, ZSCAN52 |
| ZBTB7B | ENSG00000083845 | RPS5 | S5 |
| ZBTB7B | ENSG00000084444 | KIAA1467 | KIAA1467 |
| ZBTB7B | ENSG00000084623 | EIF3I | EIF3S2, PRO2242, TRIP-1, TRIP1, eIF3-beta, eIF3-p36 |
| ZBTB7B | ENSG00000085552 | IGSF9 | FP18798, IGSF9A, Nrt1 |
| ZBTB7B | ENSG00000085644 | ZNF213 | CR53, ZKSCAN21, ZSCAN53 |
| ZBTB7B | ENSG00000085872 | CHERP | DAN16, SCAF6, SRA1 |
| ZBTB7B | ENSG00000087008 | ACOX3 | - |
| ZBTB7B | ENSG00000087074 | PPP1R15A | GADD34 |
| ZBTB7B | ENSG00000087903 | RFX2 | - |
| ZBTB7B | ENSG00000088986 | DYNLL1 | DLC1, DLC8, DNCL1, DNCLC1, LC8, LC8a, PIN, hdlc1 |
| ZBTB7B | ENSG00000089094 | KDM2B | hsa-mir-7107 |
| ZBTB7B | ENSG00000089327 | FXYD5 | DYSAD, HSPC113, IWU1, KCT1, OIT2, PRO6241, RIC |
| ZBTB7B | ENSG00000089356 | FXYD3 | hsa-mir-6887 |
| ZBTB7B | ENSG00000089639 | GMIP | ARHGAP46 |
| ZBTB7B | ENSG00000089692 | LAG3 | CD223 |
| ZBTB7B | ENSG00000089693 | MLF2 | NTN4 |
| ZBTB7B | ENSG00000090013 | BLVRB | BVRB, FLR, HEL-S-10, SDR43U1 |
| ZBTB7B | ENSG00000090097 | PCBP4 | CBP, LIP4, MCG10 |
| ZBTB7B | ENSG00000090238 | YPEL3 | - |
| ZBTB7B | ENSG00000090273 | NUDC | HNUDC, MNUDC, NPD011 |
| ZBTB7B | ENSG00000090372 | STRN4 | PPP2R6C, ZIN, zinedin |
| ZBTB7B | ENSG00000090432 | MUL1 | C1orf166, GIDE, MAPL, MULAN, RNF218 |
| ZBTB7B | ENSG00000090487 | SPG21 | ACP33, BM-019, GL010, MAST |
| ZBTB7B | ENSG00000090924 | PLEKHG2 | ARHGEF42, CLG, LDAMD |
| ZBTB7B | ENSG00000090971 | NAT14 | KLP1 |
| ZBTB7B | ENSG00000092051 | JPH4 | JP4, JPHL1 |
| ZBTB7B | ENSG00000092098 | RNF31 | HOIP, ZIBRA |
| ZBTB7B | ENSG00000093000 | NUP50 | NPAP60, NPAP60L |
| ZBTB7B | ENSG00000094914 | AAAS | AAA, AAASb, ADRACALA, ADRACALIN, ALADIN, GL003 |
| ZBTB7B | ENSG00000095066 | HOOK2 | HK2 |
| ZBTB7B | ENSG00000096384 | HSP90AB1 | D6S182, HSP84, HSP90B, HSPC2, HSPCB |
| ZBTB7B | ENSG00000096433 | ITPR3 | IP3R, IP3R3 |
| ZBTB7B | ENSG00000099194 | SCD | FADS5, MSTP008, SCD1, SCDOS, hSCD1 |
| ZBTB7B | ENSG00000099326 | MZF1 | MZF-1, MZF1B, ZFP98, ZNF42, ZSCAN6 |
| ZBTB7B | ENSG00000099330 | OCEL1 | FWP009, S863-9 |
| ZBTB7B | ENSG00000099617 | EFNA2 | ELF-1, EPLG6, HEK7-L, LERK-6, LERK6 |
| ZBTB7B | ENSG00000099622 | CIRBP | CIRP |
| ZBTB7B | ENSG00000099821 | POLRMT | APOLMT, MTRNAP, MTRPOL, h-mtRPOL |
| ZBTB7B | ENSG00000099957 | P2RX6 | P2RXL1, P2X6, P2XM |
| ZBTB7B | ENSG00000100029 | PES1 | PES |
| ZBTB7B | ENSG00000100036 | SLC35E4 | - |
| ZBTB7B | ENSG00000100142 | POLR2F | HRBP14.4, POLRF, RPABC14.4, RPABC2, RPB14.4, RPB6, RPC15 |
| ZBTB7B | ENSG00000100151 | PICK1 | PICK, PRKCABP |
| ZBTB7B | ENSG00000100218 | RSPH14 | RTDR1 |
| ZBTB7B | ENSG00000100228 | RAB36 | - |
| ZBTB7B | ENSG00000100241 | SBF1 | CMT4B3, DENND7A, MTMR5 |
| ZBTB7B | ENSG00000100242 | SUN2 | UNC84B |
| ZBTB7B | ENSG00000100246 | DNAL4 | MRMV3, PIG27 |
| ZBTB7B | ENSG00000100271 | TTLL1 | C22orf7, HS323M22B |
| ZBTB7B | ENSG00000100379 | KCTD17 | - |
| ZBTB7B | ENSG00000100395 | L3MBTL2 | H-l(3)mbt-l, L3MBT |
| ZBTB7B | ENSG00000100412 | ACO2 | ACONM, HEL-S-284, ICRD, OCA8, OPA9 |
| ZBTB7B | ENSG00000100413 | POLR3H | RPC22.9, RPC8 |
| ZBTB7B | ENSG00000100577 | GSTZ1 | GSTZ1-1, MAAI, MAI |
| ZBTB7B | ENSG00000100580 | TMED8 | FAM15B |
| ZBTB7B | ENSG00000100583 | SAMD15 | C14orf174, FAM15A |
| ZBTB7B | ENSG00000100605 | ITPK1 | ITRPK1 |
| ZBTB7B | ENSG00000100804 | PSMB5 | LMPX, MB1, X |
| ZBTB7B | ENSG00000100813 | ACIN1 | ACINUS, ACN, fSAP152 |
| ZBTB7B | ENSG00000100908 | EMC9 | C14orf122, CGI-112, FAM158A |
| ZBTB7B | ENSG00000100911 | PSME2 | hsa-mir-7703 |
| ZBTB7B | ENSG00000100918 | REC8 | HR21spB, REC8L1, Rec8p |
| ZBTB7B | ENSG00000100926 | TM9SF1 | HMP70, MP70 |
| ZBTB7B | ENSG00000101213 | PTK6 | BRK |
| ZBTB7B | ENSG00000101220 | C20orf27 | - |
| ZBTB7B | ENSG00000101222 | SPEF1 | C20orf28, CLAMP, SPEF1A |
| ZBTB7B | ENSG00000101224 | CDC25B | - |
| ZBTB7B | ENSG00000101577 | LPIN2 | - |
| ZBTB7B | ENSG00000102796 | DHRS12 | SDR40C1 |
| ZBTB7B | ENSG00000102854 | MSLN | MPF, SMRP |
| ZBTB7B | ENSG00000102858 | MGRN1 | RNF156 |
| ZBTB7B | ENSG00000102904 | TSNAXIP1 | TXI1 |
| ZBTB7B | ENSG00000102931 | ARL2BP | BART, BART1, RP66 |
| ZBTB7B | ENSG00000102934 | PLLP | PMLP, TM4SF11 |
| ZBTB7B | ENSG00000103145 | HCFC1R1 | HPIP |
| ZBTB7B | ENSG00000103152 | MPG | AAG, ADPG, APNG, CRA36.1, MDG, Mid1, PIG11, PIG16, anpg |
| ZBTB7B | ENSG00000103197 | TSC2 | LAM, PPP1R160, TSC4 |
| ZBTB7B | ENSG00000103199 | ZNF500 | ZKSCAN18, ZSCAN50 |
| ZBTB7B | ENSG00000103253 | HAGHL | - |
| ZBTB7B | ENSG00000103254 | FAM173A | C16orf24 |
| ZBTB7B | ENSG00000103260 | METRN | C16orf23, c380A1.2 |
| ZBTB7B | ENSG00000103266 | STUB1 | CHIP, HSPABP2, NY-CO-7, SCAR16, SDCCAG7, UBOX1 |
| ZBTB7B | ENSG00000103269 | RHBDL1 | RHBDL, RRP |
| ZBTB7B | ENSG00000103540 | CCP110 | CP110, Cep110 |
| ZBTB7B | ENSG00000103995 | CEP152 | MCPH4, MCPH9, SCKL5 |
| ZBTB7B | ENSG00000104129 | DNAJC17 | - |
| ZBTB7B | ENSG00000104142 | VPS18 | PEP3 |
| ZBTB7B | ENSG00000104365 | IKBKB | IKK-beta, IKK2, IKKB, IMD15, NFKBIKB |
| ZBTB7B | ENSG00000104472 | CHRAC1 | CHARC1, CHARC15, CHRAC-1, CHRAC-15, CHRAC15, YCL1 |
| ZBTB7B | ENSG00000104872 | PIH1D1 | NOP17 |
| ZBTB7B | ENSG00000104888 | SLC17A7 | BNPI, VGLUT1 |
| ZBTB7B | ENSG00000104936 | DMPK | DM, DM1, DM1PK, DMK, MDPK, MT-PK |
| ZBTB7B | ENSG00000104946 | TBC1D17 | mir-4750 |
| ZBTB7B | ENSG00000104951 | IL4I1 | FIG1, LAAO, LAO |
| ZBTB7B | ENSG00000104957 | CCDC130 | - |
| ZBTB7B | ENSG00000104960 | PTOV1 | ACID2, PTOV-1 |
| ZBTB7B | ENSG00000104969 | SGTA | SGT, alphaSGT, hSGT |
| ZBTB7B | ENSG00000104973 | MED25 | hsa-mir-6800 |
| ZBTB7B | ENSG00000105011 | ASF1B | CIA-II |
| ZBTB7B | ENSG00000105072 | C19orf44 | - |
| ZBTB7B | ENSG00000105193 | RPS16 | S16 |
| ZBTB7B | ENSG00000105220 | GPI | AMF, GNPI, NLK, PGI, PHI, SA-36, SA36 |
| ZBTB7B | ENSG00000105227 | PRX | CMT4F |
| ZBTB7B | ENSG00000105298 | CACTIN | C19orf29, NY-REN-24, fSAPc |
| ZBTB7B | ENSG00000105325 | FZR1 | CDC20C, CDH1, FZR, FZR2, HCDH, HCDH1 |
| ZBTB7B | ENSG00000105373 | GLTSCR2 | PICT-1, PICT1 |
| ZBTB7B | ENSG00000105393 | BABAM1 | C19orf62, HSPC142, MERIT40, NBA1 |
| ZBTB7B | ENSG00000105443 | CYTH2 | ARNO, CTS18, CTS18.1, PSCD2, PSCD2L, SEC7L, Sec7p-L, Sec7p-like, cytohesin-2 |
| ZBTB7B | ENSG00000105447 | GRWD1 | CDW4, GRWD, RRB1, WDR28 |
| ZBTB7B | ENSG00000105483 | CARD8 | CARDINAL, DACAR, DAKAR, NDPP, NDPP1, TUCAN |
| ZBTB7B | ENSG00000105486 | LIG1 | - |
| ZBTB7B | ENSG00000105520 | LPPR2 | LPPR2, PRG4 |
| ZBTB7B | ENSG00000105559 | PLEKHA4 | PEPP1 |
| ZBTB7B | ENSG00000105717 | PBX4 | - |
| ZBTB7B | ENSG00000105726 | ATP13A1 | ATP13A, CGI-152 |
| ZBTB7B | ENSG00000105963 | ADAP1 | CENTA1, GCS1L, p42IP4 |
| ZBTB7B | ENSG00000106003 | LFNG | SCDO3 |
| ZBTB7B | ENSG00000106009 | BRAT1 | BAAT1, C7orf27, RMFSL |
| ZBTB7B | ENSG00000106012 | IQCE | 1700028P05Rik |
| ZBTB7B | ENSG00000106077 | ABHD11 | PP1226, WBSCR21 |
| ZBTB7B | ENSG00000106290 | TAF6 | ALYUS, MGC:8964, TAF(II)70, TAF(II)80, TAF2E, TAFII-70, TAFII-80, TAFII70, TAFII80, TAFII85 |
| ZBTB7B | ENSG00000106367 | AP1S1 | AP19, CLAPS1, EKV3, MEDNIK, SIGMA1A |
| ZBTB7B | ENSG00000106477 | CEP41 | JBTS15, TSGA14 |
| ZBTB7B | ENSG00000106479 | ZNF862 | - |
| ZBTB7B | ENSG00000106484 | MEST | PEG1 |
| ZBTB7B | ENSG00000106665 | CLIP2 | CLIP, CLIP-115, CYLN2, WBSCR3, WBSCR4, WSCR3, WSCR4 |
| ZBTB7B | ENSG00000107614 | TRDMT1 | DMNT2, DNMT2, MHSAIIP, PUMET, RNMT1 |
| ZBTB7B | ENSG00000107625 | DDX50 | GU2, GUB, RH-II/GuB, mcdrh |
| ZBTB7B | ENSG00000107819 | SFXN3 | BA108L7.2, SFX3 |
| ZBTB7B | ENSG00000108010 | GLRX3 | GLRX4, GRX3, GRX4, PICOT, TXNL2, TXNL3 |
| ZBTB7B | ENSG00000108352 | RAPGEFL1 | Link-GEFII |
| ZBTB7B | ENSG00000108406 | DHX40 | ARG147, DDX40, PAD |
| ZBTB7B | ENSG00000108641 | B9D1 | B9, EPPB9, JBTS27, MKS9, MKSR1 |
| ZBTB7B | ENSG00000108883 | EFTUD2 | MFDGA, MFDM, SNRNP116, Snrp116, Snu114, U5-116KD |
| ZBTB7B | ENSG00000108963 | DPH1 | DEDSSH, DPH2L, DPH2L1, OVCA1 |
| ZBTB7B | ENSG00000109062 | SLC9A3R1 | mir-3615 |
| ZBTB7B | ENSG00000109654 | TRIM2 | CMT2R, RNF86 |
| ZBTB7B | ENSG00000109846 | CRYAB | CMD1II, CRYA2, CTPP2, CTRCT16, HEL-S-101, HSPB5, MFM2 |
| ZBTB7B | ENSG00000109881 | CCDC34 | L15, NY-REN-41, RAMA3 |
| ZBTB7B | ENSG00000110011 | DNAJC4 | DANJC4, HSPF2, MCG18 |
| ZBTB7B | ENSG00000110315 | RNF141 | ZFP26, ZNF230 |
| ZBTB7B | ENSG00000110619 | CARS | CARS1, CYSRS, MGC:11246 |
| ZBTB7B | ENSG00000110628 | SLC22A18 | BWR1A, BWSCR1A, HET, IMPT1, ITM, ORCTL2, SLC22A1L, TSSC5, p45-BWR1A |
| ZBTB7B | ENSG00000110697 | PITPNM1 | DRES9, NIR2, PITPNM, RDGB, RDGB1, RDGBA, RDGBA1, Rd9 |
| ZBTB7B | ENSG00000110713 | NUP98 | ADIR2, NUP196, NUP96 |
| ZBTB7B | ENSG00000110917 | MLEC | KIAA0152 |
| ZBTB7B | ENSG00000110955 | ATP5B | ATPMB, ATPSB, HEL-S-271 |
| ZBTB7B | ENSG00000111012 | CYP27B1 | CP2B, CYP1, CYP1alpha, CYP27B, P450c1, PDDR, VDD1, VDDR, VDDRI, VDR |
| ZBTB7B | ENSG00000111057 | KRT18 | CK-18, CYK18, K18 |
| ZBTB7B | ENSG00000111087 | GLI1 | GLI |
| ZBTB7B | ENSG00000111203 | ITFG2 | - |
| ZBTB7B | ENSG00000111319 | SCNN1A | BESC2, ENaCa, ENaCalpha, SCNEA, SCNN1 |
| ZBTB7B | ENSG00000111321 | LTBR | CD18, D12S370, LT-BETA-R, TNF-R-III, TNFCR, TNFR-RP, TNFR2-RP, TNFR3, TNFRSF3 |
| ZBTB7B | ENSG00000111331 | OAS3 | p100, p100OAS |
| ZBTB7B | ENSG00000111445 | RFC5 | RFC36 |
| ZBTB7B | ENSG00000111640 | GAPDH | G3PD, GAPD, HEL-S-162eP |
| ZBTB7B | ENSG00000111641 | NOP2 | NOL1, NOP120, NSUN1, p120 |
| ZBTB7B | ENSG00000111642 | CHD4 | CHD-4, Mi-2b, Mi2-BETA, SIHIWES |
| ZBTB7B | ENSG00000111665 | CDCA3 | GRCC8, TOME-1 |
| ZBTB7B | ENSG00000111667 | USP5 | ISOT |
| ZBTB7B | ENSG00000111669 | TPI1 | HEL-S-49, TIM, TPI, TPID |
| ZBTB7B | ENSG00000111671 | SPSB2 | GRCC9, SSB2 |
| ZBTB7B | ENSG00000111674 | ENO2 | HEL-S-279, NSE |
| ZBTB7B | ENSG00000111676 | ATN1 | B37, D12S755E, DRPLA, HRS, NOD |
| ZBTB7B | ENSG00000111678 | C12orf57 | C10, GRCC10 |
| ZBTB7B | ENSG00000111679 | PTPN6 | HCP, HCPH, HPTP1C, PTP-1C, SH-PTP1, SHP-1, SHP-1L, SHP1 |
| ZBTB7B | ENSG00000111684 | LPCAT3 | C3F, LPCAT, LPLAT 5, LPSAT, MBOAT5, OACT5, nessy |
| ZBTB7B | ENSG00000111786 | SRSF9 | SFRS9, SRp30c |
| ZBTB7B | ENSG00000112511 | PHF1 | MTF2L2, PCL1, PHF2, TDRD19C, hPHF1 |
| ZBTB7B | ENSG00000112514 | CUTA | ACHAP, C6orf82 |
| ZBTB7B | ENSG00000112530 | PACRG | GLUP, HAK005771, PARK2CRG |
| ZBTB7B | ENSG00000112759 | SLC29A1 | ENT1 |
| ZBTB7B | ENSG00000112787 | FBRSL1 | - |
| ZBTB7B | ENSG00000112972 | HMGCS1 | HMGCS |
| ZBTB7B | ENSG00000113141 | IK | - |
| ZBTB7B | ENSG00000114331 | ACAP2 | CENTB2, CNT-B2 |
| ZBTB7B | ENSG00000114353 | GNAI2 | GIP, GNAI2B, H_LUCA15.1, H_LUCA16.1 |
| ZBTB7B | ENSG00000114378 | HYAL1 | HYAL-1, LUCA1, MPS9, NAT6 |
| ZBTB7B | ENSG00000114383 | TUSC2 | C3orf11, FUS1, PAP, PDAP2 |
| ZBTB7B | ENSG00000114388 | NPRL2 | FFEVF2, NPR2, NPR2L, TUSC4 |
| ZBTB7B | ENSG00000114395 | CYB561D2 | - |
| ZBTB7B | ENSG00000114656 | KIAA1257 | - |
| ZBTB7B | ENSG00000114670 | NEK11 | - |
| ZBTB7B | ENSG00000114767 | RRP9 | RNU3IP2, U3-55K |
| ZBTB7B | ENSG00000114779 | ABHD14B | CIB, HEL-S-299 |
| ZBTB7B | ENSG00000114786 | ABHD14A-ACY1 | - |
| ZBTB7B | ENSG00000114956 | DGUOK | MTDPS3, NCPH, PEOB4, dGK |
| ZBTB7B | ENSG00000114988 | LMAN2L | MRT52, VIPL |
| ZBTB7B | ENSG00000115274 | INO80B | HMGA1L4, HMGIYL4, IES2, PAP-1BP, PAPA-1, PAPA1, ZNHIT4, hIes2 |
| ZBTB7B | ENSG00000115423 | DNAH6 | DNHL1, Dnahc6, HL-2, HL2 |
| ZBTB7B | ENSG00000115875 | SRSF7 | 9G8, AAG3, SFRS7 |
| ZBTB7B | ENSG00000115970 | THADA | ARMC13, GITA |
| ZBTB7B | ENSG00000116017 | ARID3A | BRIGHT, DRIL1, DRIL3, E2FBP1 |
| ZBTB7B | ENSG00000116138 | DNAJC16 | - |
| ZBTB7B | ENSG00000116151 | MORN1 | - |
| ZBTB7B | ENSG00000116857 | TMEM9 | DERM4, TMEM9A |
| ZBTB7B | ENSG00000117155 | SSX2IP | ADIP, hMsd1 |
| ZBTB7B | ENSG00000117650 | NEK2 | HsPK21, NEK2A, NLK1, PPP1R111, RP67 |
| ZBTB7B | ENSG00000117758 | STX12 | STX13, STX14 |
| ZBTB7B | ENSG00000118960 | HS1BP3 | ETM2, HS1-BP3 |
| ZBTB7B | ENSG00000119574 | ZBTB45 | ZNF499 |
| ZBTB7B | ENSG00000119640 | ACYP1 | ACYPE |
| ZBTB7B | ENSG00000119650 | IFT43 | C14orf179, CED3 |
| ZBTB7B | ENSG00000119660 | DPPA5P4 | - |
| ZBTB7B | ENSG00000119703 | ZC2HC1C | C14orf140, FAM164C |
| ZBTB7B | ENSG00000119718 | EIF2B2 | EIF-2Bbeta, EIF2B |
| ZBTB7B | ENSG00000120051 | CFAP58 | C10orf80, CCDC147, bA127L20.4, bA127L20.5, bA554P13.1 |
| ZBTB7B | ENSG00000121274 | PAPD5 | TRF4-2 |
| ZBTB7B | ENSG00000121281 | ADCY7 | AC7 |
| ZBTB7B | ENSG00000122042 | UBL3 | HCG-1, PNSC1 |
| ZBTB7B | ENSG00000122257 | RBBP6 | MY038, P2P-R, PACT, RBQ-1, SNAMA |
| ZBTB7B | ENSG00000122386 | ZNF205 | RhitH, ZNF210, Zfp13 |
| ZBTB7B | ENSG00000122643 | NT5C3A | NT5C3, P5'N-1, P5N-1, PN-I, POMP, PSN1, UMPH, UMPH1, cN-III, hUMP1, p36 |
| ZBTB7B | ENSG00000122735 | DNAI1 | CILD1, DIC1, ICS1, PCD |
| ZBTB7B | ENSG00000122884 | P4HA1 | P4HA |
| ZBTB7B | ENSG00000123064 | DDX54 | DP97 |
| ZBTB7B | ENSG00000123144 | C19orf43 | fSAP18 |
| ZBTB7B | ENSG00000123349 | PFDN5 | MM-1, MM1, PFD5 |
| ZBTB7B | ENSG00000123358 | NR4A1 | GFRP1, HMR, N10, NAK-1, NGFIB, NP10, NUR77, TR3 |
| ZBTB7B | ENSG00000123427 | METTL21B | FAM119B |
| ZBTB7B | ENSG00000124155 | PIGT | CGI-06, MCAHS3, NDAP, PNH2 |
| ZBTB7B | ENSG00000124160 | NCOA5 | CIA, bA465L10.6 |
| ZBTB7B | ENSG00000124588 | NQO2 | DHQV, DIA6, NMOR2, QR2 |
| ZBTB7B | ENSG00000124762 | CDKN1A | CAP20, CDKN1, CIP1, MDA-6, P21, SDI1, WAF1, p21CIP1 |
| ZBTB7B | ENSG00000125037 | EMC3 | POB, TMEM111 |
| ZBTB7B | ENSG00000125319 | C17orf53 | - |
| ZBTB7B | ENSG00000125445 | MRPS7 | MRP-S, MRP-S7, RP-S7, RPMS7, S7mt, bMRP27a |
| ZBTB7B | ENSG00000125447 | GGA3 | - |
| ZBTB7B | ENSG00000125454 | SLC25A19 | DNC, MCPHA, MUP1, THMD3, THMD4, TPC |
| ZBTB7B | ENSG00000125482 | TTF1 | TTF-1, TTF-I |
| ZBTB7B | ENSG00000125740 | FOSB | AP-1, G0S3, GOS3, GOSB |
| ZBTB7B | ENSG00000125775 | SDCBP2 | SITAC, SITAC18, ST-2 |
| ZBTB7B | ENSG00000125817 | CENPB | - |
| ZBTB7B | ENSG00000125827 | TMX4 | DJ971N18.2, PDIA14, TXNDC13 |
| ZBTB7B | ENSG00000126062 | TMEM115 | PL6 |
| ZBTB7B | ENSG00000126106 | TMEM53 | NET4 |
| ZBTB7B | ENSG00000126214 | KLC1 | KLC, KNS2, KNS2A |
| ZBTB7B | ENSG00000126368 | NR1D1 | EAR1, THRA1, THRAL, ear-1, hRev |
| ZBTB7B | ENSG00000126522 | ASL | ASAL |
| ZBTB7B | ENSG00000126602 | TRAP1 | HSP 75, HSP75, HSP90L, TRAP-1 |
| ZBTB7B | ENSG00000126787 | DLGAP5 | DLG7, HURP |
| ZBTB7B | ENSG00000126870 | WDR60 | FAP163, SRPS6, SRTD8 |
| ZBTB7B | ENSG00000127334 | DYRK2 | - |
| ZBTB7B | ENSG00000127554 | GFER | ALR, ERV1, HERV1, HPO, HPO1, HPO2, HSS |
| ZBTB7B | ENSG00000127578 | WFIKKN1 | C16orf12, RJD2, WFDC20A, WFIKKN |
| ZBTB7B | ENSG00000127580 | WDR24 | C16orf21, JFP7 |
| ZBTB7B | ENSG00000127585 | FBXL16 | C16orf22, Fbl16, c380A1.1 |
| ZBTB7B | ENSG00000128016 | ZFP36 | G0S24, GOS24, NUP475, RNF162A, TIS11, TTP, zfp-36 |
| ZBTB7B | ENSG00000128165 | ADM2 | AM2, dJ579N16.4 |
| ZBTB7B | ENSG00000128408 | RIBC2 | C22orf11, TRIB |
| ZBTB7B | ENSG00000128891 | C15orf57 | CCDC32 |
| ZBTB7B | ENSG00000128965 | CHAC1 | - |
| ZBTB7B | ENSG00000128973 | CLN6 | CLN4A, HsT18960, nclf |
| ZBTB7B | ENSG00000129007 | CALML4 | NY-BR-20 |
| ZBTB7B | ENSG00000129071 | MBD4 | MED1 |
| ZBTB7B | ENSG00000129347 | KRI1 | - |
| ZBTB7B | ENSG00000129355 | CDKN2D | INK4D, p19, p19-INK4D |
| ZBTB7B | ENSG00000129559 | NEDD8 | NEDD-8 |
| ZBTB7B | ENSG00000129562 | DAD1 | OST2 |
| ZBTB7B | ENSG00000129654 | FOXJ1 | FKHL13, HFH-4, HFH4 |
| ZBTB7B | ENSG00000129911 | KLF16 | BTEB4, DRRF, NSLP2 |
| ZBTB7B | ENSG00000130255 | RPL36 | L36 |
| ZBTB7B | ENSG00000130270 | ATP8B3 | ATPIK |
| ZBTB7B | ENSG00000130307 | USHBP1 | AIEBP, MCC2 |
| ZBTB7B | ENSG00000130312 | MRPL34 | L34mt |
| ZBTB7B | ENSG00000130402 | ACTN4 | ACTININ-4, FSGS, FSGS1 |
| ZBTB7B | ENSG00000130517 | PGPEP1 | PAP-I, PGI, PGP, PGP-I, PGPI, Pcp |
| ZBTB7B | ENSG00000130724 | CHMP2A | BC-2, BC2, CHMP2, VPS2, VPS2A |
| ZBTB7B | ENSG00000130725 | UBE2M | UBC-RS2, UBC12, hUbc12 |
| ZBTB7B | ENSG00000130731 | C16orf13 | C16orf13, JFP2 |
| ZBTB7B | ENSG00000130810 | PPAN | BXDC3, SSF, SSF-1, SSF1, SSF2 |
| ZBTB7B | ENSG00000131165 | CHMP1A | CHMP1, PCH8, PCOLN3, PRSM1, VPS46-1, VPS46A |
| ZBTB7B | ENSG00000131435 | PDLIM4 | RIL |
| ZBTB7B | ENSG00000131473 | ACLY | ACL, ATPCL, CLATP |
| ZBTB7B | ENSG00000131591 | C1orf159 | - |
| ZBTB7B | ENSG00000131652 | THOC6 | BBIS, WDR58, fSAP35 |
| ZBTB7B | ENSG00000131669 | NINJ1 | NIN1, NINJURIN |
| ZBTB7B | ENSG00000131848 | ZSCAN5A | ZNF495, ZSCAN5 |
| ZBTB7B | ENSG00000132153 | DHX30 | DDX30, RETCOR |
| ZBTB7B | ENSG00000132326 | PER2 | FASPS, FASPS1 |
| ZBTB7B | ENSG00000132535 | DLG4 | PSD95, SAP-90, SAP90 |
| ZBTB7B | ENSG00000132881 | RSG1 | C1orf89 |
| ZBTB7B | ENSG00000133265 | HSPBP1 | FES1 |
| ZBTB7B | ENSG00000133678 | TMEM254 | C10orf57, bA369J21.6 |
| ZBTB7B | ENSG00000133816 | MICAL2 | MICAL-2, MICAL2PV1, MICAL2PV2 |
| ZBTB7B | ENSG00000134020 | PEBP4 | CORK-1, CORK1, GWTM1933, HEL-S-300, PEBP-4, PRO4408, hPEBP4 |
| ZBTB7B | ENSG00000134308 | YWHAQ | 14-3-3, 1C5, HS1 |
| ZBTB7B | ENSG00000134452 | FBXO18 | FBH1, Fbx18, hFBH1 |
| ZBTB7B | ENSG00000134461 | ANKRD16 | - |
| ZBTB7B | ENSG00000134531 | EMP1 | CL-20, EMP-1, TMP |
| ZBTB7B | ENSG00000134569 | LRP4 | CLSS, CMS17, LRP-4, LRP10, MEGF7, SOST2 |
| ZBTB7B | ENSG00000134684 | YARS | CMTDIC, TYRRS, YRS, YTS |
| ZBTB7B | ENSG00000134686 | PHC2 | mir-3605 |
| ZBTB7B | ENSG00000135404 | CD63 | LAMP-3, ME491, MLA1, OMA81H, TSPAN30 |
| ZBTB7B | ENSG00000135414 | GDF11 | BMP-11, BMP11 |
| ZBTB7B | ENSG00000135424 | ITGA7 | - |
| ZBTB7B | ENSG00000135437 | RDH5 | 9cRDH, HSD17B9, RDH1, SDR9C5 |
| ZBTB7B | ENSG00000135441 | BLOC1S1 | BLOS1, BORCS1, GCN5L1, MICoA, RT14 |
| ZBTB7B | ENSG00000135446 | CDK4 | CMM3, PSK-J3 |
| ZBTB7B | ENSG00000135452 | TSPAN31 | SAS |
| ZBTB7B | ENSG00000135476 | ESPL1 | ESP1, SEPA |
| ZBTB7B | ENSG00000135587 | SMPD2 | ISC1, NSMASE, NSMASE1 |
| ZBTB7B | ENSG00000135596 | MICAL1 | MICAL, MICAL-1, NICAL |
| ZBTB7B | ENSG00000135736 | CCDC102A | - |
| ZBTB7B | ENSG00000136026 | CKAP4 | CLIMP-63, ERGIC-63, p63 |
| ZBTB7B | ENSG00000136367 | ZFHX2 | ZFH-5, ZNF409 |
| ZBTB7B | ENSG00000136425 | CIB2 | DFNB48, KIP2, USH1J |
| ZBTB7B | ENSG00000136770 | DNAJC1 | DNAJL1, ERdj1, HTJ1, MTJ1 |
| ZBTB7B | ENSG00000136807 | CDK9 | BMND15, MIRN2861 |
| ZBTB7B | ENSG00000136918 | WDR38 | - |
| ZBTB7B | ENSG00000136942 | RPL35 | L35 |
| ZBTB7B | ENSG00000136950 | ARPC5L | ARC16-2 |
| ZBTB7B | ENSG00000137103 | TMEM8B | C9orf127, NAG-5, NAG5, NGX6 |
| ZBTB7B | ENSG00000137261 | KIAA0319 | DYLX2, DYX2, NMIG |
| ZBTB7B | ENSG00000137497 | NUMA1 | NMP-22, NUMA |
| ZBTB7B | ENSG00000137814 | HAUS2 | C15orf25, CEP27, HsT17025 |
| ZBTB7B | ENSG00000137824 | RMDN3 | FAM82A2, FAM82C, RMD-3, RMD3, ptpip51 |
| ZBTB7B | ENSG00000137880 | GCHFR | GFRP, HsT16933, P35 |
| ZBTB7B | ENSG00000138670 | RASGEF1B | GPIG4 |
| ZBTB7B | ENSG00000139190 | VAMP1 | SPAX1, SYB1, VAMP-1 |
| ZBTB7B | ENSG00000139197 | PEX5 | PBD2A, PBD2B, PTS1-BP, PTS1R, PXR1, RCDP5 |
| ZBTB7B | ENSG00000139266 | MARCH9 | MARCH-IX, RNF179 |
| ZBTB7B | ENSG00000139405 | RITA1 | C12orf52, RITA |
| ZBTB7B | ENSG00000139537 | CCDC65 | CFAP250, DRC2, FAP250, NYD-SP28 |
| ZBTB7B | ENSG00000139625 | MAP3K12 | DLK, MEKK12, MUK, ZPK, ZPKP1 |
| ZBTB7B | ENSG00000139629 | GALNT6 | GALNAC-T6, GalNAcT6 |
| ZBTB7B | ENSG00000139637 | C12orf10 | Gamm1, MST024, MSTP024, MYG, MYG1 |
| ZBTB7B | ENSG00000139714 | MORN3 | - |
| ZBTB7B | ENSG00000139718 | SETD1B | KMT2G, Set1B |
| ZBTB7B | ENSG00000139725 | RHOF | ARHF, RIF |
| ZBTB7B | ENSG00000139880 | CDH24 | CDH11L |
| ZBTB7B | ENSG00000139908 | TSSK4 | C14orf20, STK22E, TSK-4, TSK4, TSSK5 |
| ZBTB7B | ENSG00000140104 | C14orf79 | - |
| ZBTB7B | ENSG00000140105 | WARS | GAMMA-2, IFI53, IFP53 |
| ZBTB7B | ENSG00000140107 | SLC25A47 | C14orf68, HDMCP, HMFN1655 |
| ZBTB7B | ENSG00000140262 | TCF12 | CRS3, HEB, HTF4, HsT17266, TCF-12, bHLHb20 |
| ZBTB7B | ENSG00000140319 | SRP14 | ALURBP |
| ZBTB7B | ENSG00000140391 | TSPAN3 | TM4-A, TM4SF8, TSPAN-3 |
| ZBTB7B | ENSG00000140488 | CELF6 | - |
| ZBTB7B | ENSG00000140905 | GCSH | GCE, NKH |
| ZBTB7B | ENSG00000140950 | TLDC1 | KIAA1609 |
| ZBTB7B | ENSG00000141013 | GAS8 | - |
| ZBTB7B | ENSG00000141030 | COPS3 | CSN3, SGN3 |
| ZBTB7B | ENSG00000141084 | RANBP10 | - |
| ZBTB7B | ENSG00000141258 | SGSM2 | RUTBC1 |
| ZBTB7B | ENSG00000141294 | LRRC46 | - |
| ZBTB7B | ENSG00000141295 | SCRN2 | Ses2 |
| ZBTB7B | ENSG00000141524 | TMC6 | EV1, EVER1, EVIN1, LAK-4P |
| ZBTB7B | ENSG00000141644 | MBD1 | CXXC3, PCM1, RFT |
| ZBTB7B | ENSG00000142538 | PTH2 | TIP39 |
| ZBTB7B | ENSG00000142541 | RPL13A | L13A, TSTA1 |
| ZBTB7B | ENSG00000142621 | FHAD1 | - |
| ZBTB7B | ENSG00000142634 | EFHD2 | SWS1 |
| ZBTB7B | ENSG00000142657 | PGD | 6PGD |
| ZBTB7B | ENSG00000142784 | WDTC1 | ADP, DCAF9 |
| ZBTB7B | ENSG00000142949 | PTPRF | BNAH2, LAR |
| ZBTB7B | ENSG00000143256 | PFDN2 | PFD2 |
| ZBTB7B | ENSG00000143324 | XPR1 | IBGC6, SYG1, X3 |
| ZBTB7B | ENSG00000143498 | TAF1A | MGC:17061, RAFI48, SL1, TAFI48 |
| ZBTB7B | ENSG00000143537 | ADAM15 | MDC15 |
| ZBTB7B | ENSG00000143549 | TPM3 | CAPM1, CFTD, HEL-189, HEL-S-82p, NEM1, OK/SW-cl.5, TM-5, TM3, TM30, TM30nm, TM5, TPMsk3, TRK, hscp30 |
| ZBTB7B | ENSG00000143570 | SLC39A1 | ZIP1, ZIRTL |
| ZBTB7B | ENSG00000143590 | EFNA3 | EFL2, EPLG3, Ehk1-L, LERK3 |
| ZBTB7B | ENSG00000143751 | SDE2 | C1orf55, dJ671D7.1 |
| ZBTB7B | ENSG00000143842 | SOX13 | ICA12, Sox-13 |
| ZBTB7B | ENSG00000143850 | PLEKHA6 | PEPP-3, PEPP3 |
| ZBTB7B | ENSG00000143933 | CALM2 | CAMII, LQT15, PHKD, PHKD2, caM |
| ZBTB7B | ENSG00000144118 | RALB | - |
| ZBTB7B | ENSG00000144589 | STK11IP | LIP1, LKB1IP, STK11IP1 |
| ZBTB7B | ENSG00000145063 | FLJ33534 | - |
| ZBTB7B | ENSG00000145632 | PLK2 | SNK, hPlk2, hSNK |
| ZBTB7B | ENSG00000146007 | ZMAT2 | Ptg-12, Snu23 |
| ZBTB7B | ENSG00000146425 | DYNLT1 | CW-1, TCTEL1, tctex-1 |
| ZBTB7B | ENSG00000146700 | SSC4D | S4D-SRCRB, SRCRB-S4D, SRCRB4D |
| ZBTB7B | ENSG00000147535 | PPAPDC1B | DPPL1, HTPAP, PPAPDC1B |
| ZBTB7B | ENSG00000147548 | WHSC1L1 | KMT3F, KMT3G, WHISTLE, WHSC1L1, pp14328 |
| ZBTB7B | ENSG00000148218 | ALAD | ALADH, PBGS |
| ZBTB7B | ENSG00000148229 | POLE3 | CHARAC17, CHRAC17, YBL1, p17 |
| ZBTB7B | ENSG00000148356 | LRSAM1 | CMT2P, RIFLE, TAL |
| ZBTB7B | ENSG00000148985 | PGAP2 | CWH43-N, FRAG1, HPMRS3, MRT17, MRT21 |
| ZBTB7B | ENSG00000149179 | C11orf49 | - |
| ZBTB7B | ENSG00000149300 | C11orf52 | - |
| ZBTB7B | ENSG00000149743 | TRPT1 | - |
| ZBTB7B | ENSG00000149761 | NUDT22 | - |
| ZBTB7B | ENSG00000149781 | FERMT3 | KIND3, MIG-2, MIG2B, UNC112C, URP2, URP2SF |
| ZBTB7B | ENSG00000149782 | PLCB3 | - |
| ZBTB7B | ENSG00000149792 | MRPL49 | C11orf4, L49mt, MRP-L49, NOF, NOF1 |
| ZBTB7B | ENSG00000149806 | FAU | FAU1, Fub1, Fubi, MNSFbeta, RPS30, S30, asr1 |
| ZBTB7B | ENSG00000149809 | TM7SF2 | ANG1, DHCR14A, NET47 |
| ZBTB7B | ENSG00000149823 | VPS51 | ANG2, ANG3, C11orf2, C11orf3, FFR |
| ZBTB7B | ENSG00000149922 | TBX6 | SCDO5 |
| ZBTB7B | ENSG00000149923 | PPP4C | PP-X, PP4, PP4C, PPH3, PPP4, PPX |
| ZBTB7B | ENSG00000149925 | ALDOA | ALDA, GSD12, HEL-S-87p |
| ZBTB7B | ENSG00000149926 | FAM57B | FP1188 |
| ZBTB7B | ENSG00000150676 | CCDC83 | CT148, HSD9 |
| ZBTB7B | ENSG00000150764 | DIXDC1 | CCD1 |
| ZBTB7B | ENSG00000150873 | C2orf50 | - |
| ZBTB7B | ENSG00000151023 | ENKUR | C10orf63, CFAP106 |
| ZBTB7B | ENSG00000152147 | GEMIN6 | - |
| ZBTB7B | ENSG00000152292 | SH2D6 | - |
| ZBTB7B | ENSG00000152475 | ZNF837 | - |
| ZBTB7B | ENSG00000152518 | ZFP36L2 | BRF2, ERF-2, ERF2, RNF162C, TIS11D |
| ZBTB7B | ENSG00000152556 | PFKM | ATP-PFK, GSD7, PFK-1, PFK1, PFKA, PFKX, PPP1R122 |
| ZBTB7B | ENSG00000153113 | CAST | BS-17, PLACK |
| ZBTB7B | ENSG00000153310 | FAM49B | BM-009, L1 |
| ZBTB7B | ENSG00000153443 | UBALD1 | FAM100A, PP11303 |
| ZBTB7B | ENSG00000153774 | CFDP1 | BCNT, BUCENTAUR, CENP-29, CP27, SWC5, Yeti, p97 |
| ZBTB7B | ENSG00000153902 | LGI4 | LGIL3 |
| ZBTB7B | ENSG00000154114 | TBCEL | El, LRRC35 |
| ZBTB7B | ENSG00000154305 | MIA3 | ARNT, D320, TANGO, TANGO1, UNQ6077 |
| ZBTB7B | ENSG00000154743 | TSEN2 | PCH2B, SEN2, SEN2L |
| ZBTB7B | ENSG00000154832 | CXXC1 | 2410002I16Rik, 5830420C16Rik, CFP1, CGBP, HsT2645, PCCX1, PHF18, SPP1, ZCGPC1, hCGBP |
| ZBTB7B | ENSG00000155980 | KIF5A | D12S1889, MY050, NKHC, SPG10 |
| ZBTB7B | ENSG00000156453 | PCDH1 | PC42, PCDH42 |
| ZBTB7B | ENSG00000156858 | PRR14 | - |
| ZBTB7B | ENSG00000156860 | FBRS | FBS, FBS1 |
| ZBTB7B | ENSG00000157593 | SLC35B2 | mir-4647 |
| ZBTB7B | ENSG00000157637 | SLC38A10 | PP1744 |
| ZBTB7B | ENSG00000157653 | C9orf43 | - |
| ZBTB7B | ENSG00000157873 | TNFRSF14 | ATAR, CD270, HVEA, HVEM, LIGHTR, TR2 |
| ZBTB7B | ENSG00000157911 | PEX10 | NALD, PBD6A, PBD6B, RNF69 |
| ZBTB7B | ENSG00000157916 | RER1 | - |
| ZBTB7B | ENSG00000157933 | SKI | SGS, SKV |
| ZBTB7B | ENSG00000158008 | EXTL1 | EXTL |
| ZBTB7B | ENSG00000158014 | SLC30A2 | PP12488, TNZD, ZNT2, ZnT-2 |
| ZBTB7B | ENSG00000158106 | RHPN1 | ODF5, RHOPHILIN, RHPN |
| ZBTB7B | ENSG00000158109 | TPRG1L | FAM79A, h-mover, mover |
| ZBTB7B | ENSG00000158158 | CNNM4 | ACDP4 |
| ZBTB7B | ENSG00000158480 | SPATA2 | PD1, PPP1R145, tamo |
| ZBTB7B | ENSG00000158792 | SPATA2L | C16orf76, tamo |
| ZBTB7B | ENSG00000158793 | NIT1 | - |
| ZBTB7B | ENSG00000159111 | MRPL10 | L10MT, MRP-L10, MRP-L8, MRPL8, RPML8 |
| ZBTB7B | ENSG00000159214 | CCDC24 | - |
| ZBTB7B | ENSG00000159239 | C2orf81 | hCG40743 |
| ZBTB7B | ENSG00000159335 | PTMS | ParaT |
| ZBTB7B | ENSG00000159433 | STARD9 | KIF16A |
| ZBTB7B | ENSG00000159579 | RSPRY1 | SEMDFA |
| ZBTB7B | ENSG00000159674 | SPON2 | DIL-1, DIL1, M-SPONDIN, MINDIN |
| ZBTB7B | ENSG00000159720 | ATP6V0D1 | ATP6D, ATP6DV, P39, VATX, VMA6, VPATPD |
| ZBTB7B | ENSG00000160050 | CCDC28B | - |
| ZBTB7B | ENSG00000160051 | IQCC | - |
| ZBTB7B | ENSG00000160055 | TMEM234 | AASL548, C1orf91, PRO1105, RP4-622L5, dJ622L5.7 |
| ZBTB7B | ENSG00000160113 | NR2F6 | EAR-2, EAR2, ERBAL2 |
| ZBTB7B | ENSG00000160188 | RSPH1 | CT79, RSP44, RSPH10A, TSA2, TSGA2 |
| ZBTB7B | ENSG00000160190 | SLC37A1 | G3PP |
| ZBTB7B | ENSG00000160293 | VAV2 | VAV-2 |
| ZBTB7B | ENSG00000160326 | SLC2A6 | GLUT6, GLUT9, HSA011372 |
| ZBTB7B | ENSG00000160401 | C9orf117 | C9orf117 |
| ZBTB7B | ENSG00000160404 | TOR2A | TORP1 |
| ZBTB7B | ENSG00000160460 | SPTBN4 | QV, SPNB4, SPTBN3 |
| ZBTB7B | ENSG00000160570 | DEDD2 | FLAME-3, FLAME3 |
| ZBTB7B | ENSG00000160584 | SIK3 | L19, QSK, SIK-3 |
| ZBTB7B | ENSG00000160972 | PPP1R16A | MYPT3 |
| ZBTB7B | ENSG00000160973 | FOXH1 | FAST-1, FAST1 |
| ZBTB7B | ENSG00000161091 | MFSD12 | C19orf28, PP3501 |
| ZBTB7B | ENSG00000161149 | TUBA3FP | - |
| ZBTB7B | ENSG00000161533 | ACOX1 | ACOX, PALMCOX, SCOX |
| ZBTB7B | ENSG00000161714 | PLCD3 | PLC-delta-3 |
| ZBTB7B | ENSG00000161980 | POLR3K | C11, C11-RNP3, My010, RPC10, RPC11, RPC12.5 |
| ZBTB7B | ENSG00000161981 | SNRNP25 | C16orf33 |
| ZBTB7B | ENSG00000161996 | WDR90 | C16orf15, C16orf16, C16orf17, C16orf18, C16orf19 |
| ZBTB7B | ENSG00000161999 | JMJD8 | C16orf20, PP14397 |
| ZBTB7B | ENSG00000162004 | CCDC78 | C16orf25, CNM4, JFP10, hsCCDC78 |
| ZBTB7B | ENSG00000162069 | CCDC64B | BICDR-2, BICDR2, CCDC64B |
| ZBTB7B | ENSG00000162076 | FLYWCH2 | - |
| ZBTB7B | ENSG00000162188 | GNG3 | - |
| ZBTB7B | ENSG00000162227 | TAF6L | PAF65A |
| ZBTB7B | ENSG00000162241 | SLC25A45 | - |
| ZBTB7B | ENSG00000162298 | SYVN1 | hsa-mir-6751 |
| ZBTB7B | ENSG00000162510 | MATN1 | CMP, CRTM |
| ZBTB7B | ENSG00000162517 | PEF1 | ABP32, PEF1A |
| ZBTB7B | ENSG00000162571 | TTLL10 | TTLL5 |
| ZBTB7B | ENSG00000162585 | C1orf86 | C1orf86, FP7162 |
| ZBTB7B | ENSG00000162755 | KLHDC9 | KARCA1 |
| ZBTB7B | ENSG00000162869 | PPP1R21 | CCDC128, KLRAQ1 |
| ZBTB7B | ENSG00000162929 | KIAA1841 | - |
| ZBTB7B | ENSG00000163399 | ATP1A1 | - |
| ZBTB7B | ENSG00000163584 | RPL22L1 | - |
| ZBTB7B | ENSG00000163738 | MTHFD2L | - |
| ZBTB7B | ENSG00000163913 | IFT122 | CED, CED1, SPG, WDR10, WDR10p, WDR140 |
| ZBTB7B | ENSG00000163956 | LRPAP1 | A2MRAP, A2RAP, HBP44, MRAP, MYP23, RAP, alpha-2-MRAP |
| ZBTB7B | ENSG00000164050 | PLXNB1 | PLEXIN-B1, PLXN5, SEP |
| ZBTB7B | ENSG00000164877 | MICALL2 | JRAB, MICAL-L2 |
| ZBTB7B | ENSG00000164880 | INTS1 | INT1, NET28 |
| ZBTB7B | ENSG00000164970 | FAM219A | C9orf25 |
| ZBTB7B | ENSG00000165280 | VCP | ALS14, CMT2Y, HEL-220, HEL-S-70, IBMPFD, IBMPFD1, TERA, p97 |
| ZBTB7B | ENSG00000165282 | PIGO | HPMRS2 |
| ZBTB7B | ENSG00000165283 | STOML2 | HSPC108, SLP-2 |
| ZBTB7B | ENSG00000165322 | ARHGAP12 | - |
| ZBTB7B | ENSG00000165802 | NSMF | hsa-mir-7114 |
| ZBTB7B | ENSG00000166046 | TCP11L2 | - |
| ZBTB7B | ENSG00000166133 | RPUSD2 | C15orf19, C18B11 |
| ZBTB7B | ENSG00000166140 | ZFYVE19 | ANCHR, MPFYVE |
| ZBTB7B | ENSG00000166166 | TRMT61A | C14orf172, GCD14, Gcd14p, TRM61, hTRM61 |
| ZBTB7B | ENSG00000166170 | BAG5 | BAG-5 |
| ZBTB7B | ENSG00000166246 | C16orf71 | - |
| ZBTB7B | ENSG00000166411 | IDH3A | - |
| ZBTB7B | ENSG00000166455 | C16orf46 | - |
| ZBTB7B | ENSG00000166484 | MAPK7 | BMK1, ERK4, ERK5, PRKM7 |
| ZBTB7B | ENSG00000166508 | MCM7 | CDC47, MCM2, P1.1-MCM3, P1CDC47, P85MCM, PNAS146, PPP1R104 |
| ZBTB7B | ENSG00000166881 | TMEM194A | TMEM194, TMEM194A |
| ZBTB7B | ENSG00000166888 | STAT6 | D12S1644, IL-4-STAT, STAT6B, STAT6C |
| ZBTB7B | ENSG00000166963 | MAP1A | MAP1L, MTAP1A |
| ZBTB7B | ENSG00000166974 | MAPRE2 | CSCSC2, EB1, EB2, RP1 |
| ZBTB7B | ENSG00000166997 | CNPY4 | PRAT4B |
| ZBTB7B | ENSG00000167065 | DUSP18 | DSP18, DUSP20, LMWDSP20 |
| ZBTB7B | ENSG00000167081 | PBX3 | - |
| ZBTB7B | ENSG00000167094 | TTC16 | - |
| ZBTB7B | ENSG00000167131 | CCDC103 | - |
| ZBTB7B | ENSG00000167323 | STIM1 | - |
| ZBTB7B | ENSG00000167470 | MIDN | - |
| ZBTB7B | ENSG00000167523 | SPATA33 | C16orf55 |
| ZBTB7B | ENSG00000167565 | SERTAD3 | RBT1 |
| ZBTB7B | ENSG00000167701 | GPT | AAT1, ALT1, GPT1 |
| ZBTB7B | ENSG00000167702 | KIFC2 | - |
| ZBTB7B | ENSG00000167721 | TSR1 | - |
| ZBTB7B | ENSG00000167733 | HSD11B1L | 11-DH3, 11-beta-HSD3, HSD3, SCDR10, SCDR10B, SDR26C2 |
| ZBTB7B | ENSG00000167792 | NDUFV1 | CI-51K, CI51KD, UQOR1 |
| ZBTB7B | ENSG00000167797 | CDK2AP2 | DOC-1R, p14 |
| ZBTB7B | ENSG00000167895 | TMC8 | EV2, EVER2, EVIN2 |
| ZBTB7B | ENSG00000167962 | ZNF598 | - |
| ZBTB7B | ENSG00000167967 | E4F1 | E4F |
| ZBTB7B | ENSG00000167971 | CASKIN1 | ANKS5A |
| ZBTB7B | ENSG00000168000 | BSCL2 | GNG3LG, HMN5, PELD, SPG17 |
| ZBTB7B | ENSG00000168014 | C2CD3 | OFD14 |
| ZBTB7B | ENSG00000168026 | TTC21A | hsa-mir-6822 |
| ZBTB7B | ENSG00000168066 | SF1 | BBP, D11S636, MBBP, ZCCHC25, ZFM1, ZNF162 |
| ZBTB7B | ENSG00000168096 | ANKS3 | - |
| ZBTB7B | ENSG00000168209 | DDIT4 | Dig2, REDD-1, REDD1 |
| ZBTB7B | ENSG00000168216 | LMBRD1 | C6orf209, LMBD1, MAHCF, NESI |
| ZBTB7B | ENSG00000168256 | NKIRAS2 | KBRAS2, kappaB-Ras2 |
| ZBTB7B | ENSG00000168259 | DNAJC7 | DJ11, DJC7, TPR2, TTC2 |
| ZBTB7B | ENSG00000168490 | PHYHIP | DYRK1AP3, PAHX-AP, PAHXAP1 |
| ZBTB7B | ENSG00000168495 | POLR3D | BN51T, RPC4, RPC53, TSBN51 |
| ZBTB7B | ENSG00000168569 | TMEM223 | - |
| ZBTB7B | ENSG00000168778 | TCTN2 | C12orf38, JBTS24, MKS8, TECT2 |
| ZBTB7B | ENSG00000168924 | LETM1 | - |
| ZBTB7B | ENSG00000169231 | THBS3 | TSP3 |
| ZBTB7B | ENSG00000169813 | HNRNPF | HNRPF, OK/SW-cl.23, mcs94-1 |
| ZBTB7B | ENSG00000169885 | CALML6 | CAGLP |
| ZBTB7B | ENSG00000169951 | ZNF764 | - |
| ZBTB7B | ENSG00000169955 | ZNF747 | - |
| ZBTB7B | ENSG00000169957 | ZNF768 | - |
| ZBTB7B | ENSG00000170265 | ZNF282 | HUB1 |
| ZBTB7B | ENSG00000170276 | HSPB2 | - |
| ZBTB7B | ENSG00000170291 | ELP5 | C17orf81, DERP6, HSPC002, MST071, MSTP071 |
| ZBTB7B | ENSG00000170345 | FOS | AP-1, C-FOS, p55 |
| ZBTB7B | ENSG00000170374 | SP7 | OI11, OI12, OSX, osterix |
| ZBTB7B | ENSG00000170412 | GPRC5C | RAIG-3, RAIG3 |
| ZBTB7B | ENSG00000170421 | KRT8 | CARD2, CK-8, CK8, CYK8, K2C8, K8, KO |
| ZBTB7B | ENSG00000170469 | SPATA24 | CCDC161, T6441 |
| ZBTB7B | ENSG00000170855 | TRIAP1 | HSPC132, MDM35, P53CSV, WF-1 |
| ZBTB7B | ENSG00000171223 | JUNB | AP-1 |
| ZBTB7B | ENSG00000171443 | ZNF524 | - |
| ZBTB7B | ENSG00000171475 | WIPF2 | WICH, WIRE |
| ZBTB7B | ENSG00000171552 | BCL2L1 | BCL-XL/S, BCL2L, BCLX, Bcl-X, PPP1R52 |
| ZBTB7B | ENSG00000171574 | ZNF584 | - |
| ZBTB7B | ENSG00000171953 | ATPAF2 | ATP12, ATP12p, LP3663, MC5DN1 |
| ZBTB7B | ENSG00000171962 | LRRC48 | CFAP134, LRRC48 |
| ZBTB7B | ENSG00000172009 | THOP1 | EP24.15, MEPD_HUMAN, MP78, TOP |
| ZBTB7B | ENSG00000172053 | QARS | hsa-mir-6890 |
| ZBTB7B | ENSG00000172269 | DPAGT1 | ALG7, CDG-Ij, CDG1J, CMS13, CMSTA2, D11S366, DGPT, DPAGT, DPAGT2, G1PT, GPT, UAGT, UGAT |
| ZBTB7B | ENSG00000172273 | HINFP | HiNF-P, MIZF, ZNF743 |
| ZBTB7B | ENSG00000172336 | POP7 | 0610037N12Rik, RPP2, RPP20 |
| ZBTB7B | ENSG00000172361 | CFAP53 | CCDC11, HTX6 |
| ZBTB7B | ENSG00000172366 | FAM195A | - |
| ZBTB7B | ENSG00000172375 | C2CD2L | DLNB23, TMEM24 |
| ZBTB7B | ENSG00000172460 | PRSS30P | Disp, TMPRSS8, TMPRSS8P |
| ZBTB7B | ENSG00000172466 | ZNF24 | KOX17, RSG-A, ZNF191, ZSCAN3, Zfp191 |
| ZBTB7B | ENSG00000172613 | RAD9A | RAD9 |
| ZBTB7B | ENSG00000172771 | EFCAB12 | C3orf25 |
| ZBTB7B | ENSG00000172794 | RAB37 | - |
| ZBTB7B | ENSG00000172830 | SSH3 | SSH3L |
| ZBTB7B | ENSG00000172932 | ANKRD13D | - |
| ZBTB7B | ENSG00000173153 | ESRRA | ERR1, ERRa, ERRalpha, ESRL1, NR3B1 |
| ZBTB7B | ENSG00000173171 | MTX1 | MTX, MTXN |
| ZBTB7B | ENSG00000173214 | KIAA1919 | KIAA1919, NaGLT1 |
| ZBTB7B | ENSG00000173264 | GPR137 | C11orf4, GPR137A, TM7SF1L1 |
| ZBTB7B | ENSG00000173295 | FAM86B3P | - |
| ZBTB7B | ENSG00000173457 | PPP1R14B | PHI-1, PLCB3N, PNG, SOM172 |
| ZBTB7B | ENSG00000173486 | FKBP2 | FKBP-13, PPIase |
| ZBTB7B | ENSG00000173511 | VEGFB | VEGFL, VRF |
| ZBTB7B | ENSG00000173812 | EIF1 | A121, EIF-1, EIF1A, ISO1, SUI1 |
| ZBTB7B | ENSG00000173848 | NET1 | ARHGEF8, NET1A |
| ZBTB7B | ENSG00000173926 | MARCH3 | MARCH-III, RNF173 |
| ZBTB7B | ENSG00000173928 | SWSAP1 | C19orf39, SWS1AP1, ZSWIM7AP1 |
| ZBTB7B | ENSG00000173933 | RBM4 | LARK, RBM4A, ZCCHC21, ZCRB3A |
| ZBTB7B | ENSG00000173992 | CCS | - |
| ZBTB7B | ENSG00000174276 | ZNHIT2 | C11orf5, FON |
| ZBTB7B | ENSG00000174483 | BBS1 | - |
| ZBTB7B | ENSG00000174516 | PELI3 | - |
| ZBTB7B | ENSG00000174547 | MRPL11 | CGI-113, L11MT, MRP-L11 |
| ZBTB7B | ENSG00000174586 | ZNF497 | - |
| ZBTB7B | ENSG00000174684 | B3GNT1 | B3GN-T1, B3GNT1, B3GNT6, BETA3GNTI, MDDGA13, iGAT, iGNT |
| ZBTB7B | ENSG00000174744 | BRMS1 | - |
| ZBTB7B | ENSG00000174791 | RIN1 | - |
| ZBTB7B | ENSG00000174851 | YIF1A | 54TM, FinGER7, YIF1, YIF1P |
| ZBTB7B | ENSG00000174871 | CNIH2 | CNIH-2, Cnil |
| ZBTB7B | ENSG00000174903 | RAB1B | - |
| ZBTB7B | ENSG00000174915 | PTDSS2 | PSS2 |
| ZBTB7B | ENSG00000174917 | C19orf70 | MIC13, P117, QIL1 |
| ZBTB7B | ENSG00000174996 | KLC2 | - |
| ZBTB7B | ENSG00000175203 | DCTN2 | DCTN50, DYNAMITIN, HEL-S-77, RBP50 |
| ZBTB7B | ENSG00000175279 | APITD1 | APITD1, CENP-S, FAAP16, MHF1 |
| ZBTB7B | ENSG00000175567 | UCP2 | BMIQ4, SLC25A8, UCPH |
| ZBTB7B | ENSG00000175662 | TOM1L2 | - |
| ZBTB7B | ENSG00000175711 | B3GNTL1 | 3-Gn-T8, B3GNT8, BGnT-8, beta-1, beta3Gn-T8, beta3GnTL1 |
| ZBTB7B | ENSG00000175826 | CTDNEP1 | DULLARD, HSA011916, NET56 |
| ZBTB7B | ENSG00000176095 | IP6K1 | IHPK1, PiUS |
| ZBTB7B | ENSG00000176182 | MYPOP | P42pop |
| ZBTB7B | ENSG00000176473 | WDR25 | C14orf67 |
| ZBTB7B | ENSG00000176903 | PNMA1 | MA1 |
| ZBTB7B | ENSG00000177045 | SIX5 | BOR2, DMAHP |
| ZBTB7B | ENSG00000177112 | MRVI1-AS1 | - |
| ZBTB7B | ENSG00000177542 | SLC25A22 | EIEE3, GC-1, GC1, NET44 |
| ZBTB7B | ENSG00000177595 | PIDD1 | LRDD, PIDD |
| ZBTB7B | ENSG00000177600 | RPLP2 | D11S2243E, LP2, P2, RPP2 |
| ZBTB7B | ENSG00000177666 | PNPLA2 | 1110001C14Rik, ATGL, FP17548, PEDF-R, TTS-2.2, TTS2, iPLA2zeta |
| ZBTB7B | ENSG00000177674 | AGTRAP | ATRAP |
| ZBTB7B | ENSG00000177685 | CRACR2B | EFCAB4A |
| ZBTB7B | ENSG00000177697 | CD151 | GP27, MER2, PETA-3, RAPH, SFA1, TSPAN24 |
| ZBTB7B | ENSG00000177700 | POLR2L | RBP10, RPABC5, RPB10, RPB10beta, RPB7.6, hRPB7.6 |
| ZBTB7B | ENSG00000177943 | MAMDC4 | AEGP |
| ZBTB7B | ENSG00000177994 | C2orf73 | - |
| ZBTB7B | ENSG00000178035 | IMPDH2 | IMPD2, IMPDH-II |
| ZBTB7B | ENSG00000178057 | NDUFAF3 | 2P1, C3orf60, E3-3 |
| ZBTB7B | ENSG00000178149 | DALRD3 | - |
| ZBTB7B | ENSG00000178150 | ZNF114 | - |
| ZBTB7B | ENSG00000178209 | PLEC | EBS1, EBSMD, EBSND, EBSO, EBSOG, EBSPA, HD1, LGMD2Q, PCN, PLEC1, PLEC1b, PLTN |
| ZBTB7B | ENSG00000178252 | WDR6 | - |
| ZBTB7B | ENSG00000178467 | P4HTM | EGLN4, HIFPH4, P4H-TM, PH-4, PH4, PHD4 |
| ZBTB7B | ENSG00000178685 | PARP10 | ARTD10 |
| ZBTB7B | ENSG00000178719 | GRINA | HNRGW, LFG1, NMDARA1, TMBIM3 |
| ZBTB7B | ENSG00000178821 | TMEM52 | - |
| ZBTB7B | ENSG00000178980 | SEPW1 | SEPW1, selW |
| ZBTB7B | ENSG00000178999 | AURKB | AIK2, AIM-1, AIM1, ARK2, AurB, IPL1, PPP1R48, STK12, STK5, aurkb-sv1, aurkb-sv2 |
| ZBTB7B | ENSG00000179029 | TMEM107 | GRVS638, PRO1268 |
| ZBTB7B | ENSG00000179134 | SAMD4B | SMGB, Smaug2 |
| ZBTB7B | ENSG00000179151 | EDC3 | LSM16, MRT50, YJDC, YJEFN2 |
| ZBTB7B | ENSG00000179292 | TMEM151A | TMEM151 |
| ZBTB7B | ENSG00000179335 | CLK3 | PHCLK3, PHCLK3/152 |
| ZBTB7B | ENSG00000179361 | ARID3B | BDP, DRIL2 |
| ZBTB7B | ENSG00000179564 | LSMEM2 | C3orf45 |
| ZBTB7B | ENSG00000179933 | C14orf119 | - |
| ZBTB7B | ENSG00000179943 | FIZ1 | ZNF798 |
| ZBTB7B | ENSG00000179965 | ZNF771 | DSC43 |
| ZBTB7B | ENSG00000180096 | SEPT1 | DIFF6, LARP, PNUTL3, SEP1 |
| ZBTB7B | ENSG00000180881 | CAPS2 | UG0636c06 |
| ZBTB7B | ENSG00000180902 | D2HGDH | D2HGD |
| ZBTB7B | ENSG00000180921 | FAM83H | AI3 |
| ZBTB7B | ENSG00000180979 | LRRC57 | - |
| ZBTB7B | ENSG00000181027 | FKRP | LGMD2I, MDC1C, MDDGA5, MDDGB5, MDDGC5 |
| ZBTB7B | ENSG00000181085 | MAPK15 | ERK7, ERK8 |
| ZBTB7B | ENSG00000181513 | ACBD4 | HMFT0700 |
| ZBTB7B | ENSG00000181649 | PHLDA2 | BRW1C, BWR1C, HLDA2, IPL, TSSC3 |
| ZBTB7B | ENSG00000181885 | CLDN7 | CEPTRL2, CLDN-7, CPETRL2, Hs.84359, claudin-1 |
| ZBTB7B | ENSG00000181896 | ZNF101 | HZF12 |
| ZBTB7B | ENSG00000182095 | TNRC18 | CAGL79, TNRC18A |
| ZBTB7B | ENSG00000182179 | UBA7 | mir-5193 |
| ZBTB7B | ENSG00000182324 | KCNJ14 | IRK4, KIR2.4 |
| ZBTB7B | ENSG00000182450 | KCNK4 | K2p4.1, TRAAK, TRAAK1 |
| ZBTB7B | ENSG00000182544 | MFSD5 | hsMOT2 |
| ZBTB7B | ENSG00000182768 | NGRN | DSC92 |
| ZBTB7B | ENSG00000182791 | CCDC87 | - |
| ZBTB7B | ENSG00000182809 | CRIP2 | CRIP, CRP2, ESP1 |
| ZBTB7B | ENSG00000182866 | LCK | IMD22, LSK, YT16, p56lck, pp58lck |
| ZBTB7B | ENSG00000182896 | TMEM95 | UNQ9390 |
| ZBTB7B | ENSG00000182950 | ODF3L1 | - |
| ZBTB7B | ENSG00000182979 | MTA1 | - |
| ZBTB7B | ENSG00000183208 | GDPGP1 | C15orf58, VTC2 |
| ZBTB7B | ENSG00000183971 | NPW | L8, L8C, PPL8, PPNPW |
| ZBTB7B | ENSG00000184007 | PTP4A2 | HH13, HH7-2, HU-PP-1, OV-1, PRL-2, PRL2, PTP4A, PTPCAAX2, ptp-IV1a, ptp-IV1b |
| ZBTB7B | ENSG00000184009 | ACTG1 | ACT, ACTG, BRWS2, DFNA20, DFNA26, HEL-176 |
| ZBTB7B | ENSG00000184154 | LRTOMT | CFAP111, DFNB63, LRRC51 |
| ZBTB7B | ENSG00000184224 | C11orf72 | - |
| ZBTB7B | ENSG00000184436 | THAP7 | - |
| ZBTB7B | ENSG00000184675 | AMER1 | FAM123B, OSCS, WTX |
| ZBTB7B | ENSG00000184697 | CLDN6 | - |
| ZBTB7B | ENSG00000184925 | LCN12 | - |
| ZBTB7B | ENSG00000184939 | ZFP90 | FIK, NK10, ZNF756, zfp-90 |
| ZBTB7B | ENSG00000185033 | SEMA4B | SEMAC, SemC |
| ZBTB7B | ENSG00000185043 | CIB1 | CIB, CIBP, KIP1, PRKDCIP, SIP2-28 |
| ZBTB7B | ENSG00000185101 | ANO9 | PIG5, TMEM16J, TP53I5 |
| ZBTB7B | ENSG00000185250 | PPIL6 | PPIase, RSPH12, bA425D10.6, dJ919F19.1 |
| ZBTB7B | ENSG00000185298 | CCDC137 | RaRF |
| ZBTB7B | ENSG00000185324 | CDK10 | PISSLRE |
| ZBTB7B | ENSG00000185332 | TMEM105 | - |
| ZBTB7B | ENSG00000185344 | ATP6V0A2 | A2, ARCL, ARCL2A, ATP6A2, ATP6N1D, J6B7, RTF, STV1, TJ6, TJ6M, TJ6S, VPH1, WSS |
| ZBTB7B | ENSG00000185345 | PARK2 | AR-JP, LPRS2, PDJ, PRKN |
| ZBTB7B | ENSG00000185347 | C14orf80 | - |
| ZBTB7B | ENSG00000185359 | HGS | HRS |
| ZBTB7B | ENSG00000185453 | C19orf68 | - |
| ZBTB7B | ENSG00000185475 | TMEM179B | hsa-mir-6748 |
| ZBTB7B | ENSG00000185499 | MUC1 | ADMCKD, ADMCKD1, CA 15-3, CD227, EMA, H23AG, KL-6, MAM6, MCD, MCKD, MCKD1, MUC-1, MUC-1/SEC, MUC-1/X, MUC1/ZD, PEM, PEMT, PUM |
| ZBTB7B | ENSG00000185527 | PDE6G | PDEG, RP57 |
| ZBTB7B | ENSG00000185567 | AHNAK2 | C14orf78 |
| ZBTB7B | ENSG00000185614 | FAM212A | C3orf54, INKA1 |
| ZBTB7B | ENSG00000185800 | DMWD | D19S593E, DMR-N9, DMRN9, gene59 |
| ZBTB7B | ENSG00000185875 | THNSL1 | TSH1 |
| ZBTB7B | ENSG00000186056 | MATN1-AS1 | - |
| ZBTB7B | ENSG00000186153 | WWOX | D16S432E, EIEE28, FOR, FRA16D, HHCMA56, PRO0128, SCAR12, SDR41C1, WOX1 |
| ZBTB7B | ENSG00000186185 | KIF18B | - |
| ZBTB7B | ENSG00000186283 | TOR3A | ADIR, ADIR2 |
| ZBTB7B | ENSG00000186301 | MST1P2 | - |
| ZBTB7B | ENSG00000186496 | ZNF396 | ZSCAN14 |
| ZBTB7B | ENSG00000186710 | CCDC42B | CCDC42B, MIA2 |
| ZBTB7B | ENSG00000186792 | HYAL3 | HYAL-3, LUCA-3, LUCA3 |
| ZBTB7B | ENSG00000186862 | PDZD7 | DFNB57, PDZK7 |
| ZBTB7B | ENSG00000187024 | PTRH1 | C9orf115, PTH1 |
| ZBTB7B | ENSG00000187147 | RNF220 | C1orf164 |
| ZBTB7B | ENSG00000187266 | EPOR | EPO-R |
| ZBTB7B | ENSG00000187492 | CDHR4 | CDH29, PRO34300 |
| ZBTB7B | ENSG00000187556 | NANOS3 | NANOS1L, NOS3, ZC2HC12C |
| ZBTB7B | ENSG00000187905 | LRRC74B | - |
| ZBTB7B | ENSG00000187954 | CYHR1 | CHRP |
| ZBTB7B | ENSG00000187997 | C17orf99 | UNQ464 |
| ZBTB7B | ENSG00000188015 | S100A3 | S100E |
| ZBTB7B | ENSG00000188112 | C6orf132 | bA7K24.2 |
| ZBTB7B | ENSG00000188277 | C15orf62 | - |
| ZBTB7B | ENSG00000188372 | ZP3 | ZP3A, ZP3B, ZPC, Zp-3 |
| ZBTB7B | ENSG00000188486 | H2AFX | H2A.X, H2A/X, H2AX |
| ZBTB7B | ENSG00000188596 | CFAP54 | C12orf55, C12orf63 |
| ZBTB7B | ENSG00000188643 | S100A16 | AAG13, DT1P1A7, S100F |
| ZBTB7B | ENSG00000188786 | MTF1 | MTF-1, ZRF |
| ZBTB7B | ENSG00000188878 | FBF1 | Alb, FBF-1 |
| ZBTB7B | ENSG00000189077 | TMEM120A | NET29, TMPIT |
| ZBTB7B | ENSG00000189157 | FAM47E | - |
| ZBTB7B | ENSG00000196154 | S100A4 | 18A2, 42A, CAPL, FSP1, MTS1, P9KA, PEL98 |
| ZBTB7B | ENSG00000196235 | SUPT5H | SPT5, SPT5H, Tat-CT1 |
| ZBTB7B | ENSG00000196391 | ZNF774 | - |
| ZBTB7B | ENSG00000196396 | PTPN1 | PTP1B |
| ZBTB7B | ENSG00000196408 | NOXO1 | P41NOX, P41NOXA, P41NOXB, P41NOXC, SH3PXD5, SNX28 |
| ZBTB7B | ENSG00000196420 | S100A5 | S100D |
| ZBTB7B | ENSG00000196476 | C20orf96 | dJ1103G7.2 |
| ZBTB7B | ENSG00000196497 | IPO4 | Imp4 |
| ZBTB7B | ENSG00000196498 | NCOR2 | CTG26, N-CoR2, SMAP270, SMRT, SMRTE, SMRTE-tau, TNRC14, TRAC, TRAC-1, TRAC1 |
| ZBTB7B | ENSG00000196517 | SLC6A9 | GLYT1 |
| ZBTB7B | ENSG00000196535 | MYO18A | MAJN, MYSPDZ, SPR210 |
| ZBTB7B | ENSG00000196642 | RABL6 | C9orf86, PARF, RBEL1, pp8875 |
| ZBTB7B | ENSG00000196704 | AMZ2 | - |
| ZBTB7B | ENSG00000196754 | S100A2 | CAN19, S100L |
| ZBTB7B | ENSG00000196961 | AP2A1 | ADTAA, AP2-ALPHA, CLAPA1 |
| ZBTB7B | ENSG00000197019 | SERTAD1 | SEI1, TRIP-Br1 |
| ZBTB7B | ENSG00000197024 | ZNF398 | P51, P71, ZER6 |
| ZBTB7B | ENSG00000197077 | KIAA1671 | - |
| ZBTB7B | ENSG00000197119 | SLC25A29 | C14orf69, CACL, ORNT3 |
| ZBTB7B | ENSG00000197168 | NEK5 | - |
| ZBTB7B | ENSG00000197208 | SLC22A4 | DFNB60, OCTN1 |
| ZBTB7B | ENSG00000197251 | LINC00336 | C6orf227, NCRNA00336 |
| ZBTB7B | ENSG00000197283 | SYNGAP1 | mir-5004 |
| ZBTB7B | ENSG00000197329 | PELI1 | - |
| ZBTB7B | ENSG00000197405 | C5AR1 | C5A, C5AR, C5R1, CD88 |
| ZBTB7B | ENSG00000197555 | SIPA1L1 | E6TP1 |
| ZBTB7B | ENSG00000197580 | BCO2 | B-DIOX-II, BCDO2 |
| ZBTB7B | ENSG00000197956 | S100A6 | 2A9, 5B10, CABP, CACY, PRA |
| ZBTB7B | ENSG00000197958 | RPL12 | L12 |
| ZBTB7B | ENSG00000197980 | LEKR1 | - |
| ZBTB7B | ENSG00000198218 | QRICH1 | - |
| ZBTB7B | ENSG00000198356 | ASNA1 | ARSA-I, ARSA1, ASNA-I, GET3, TRC40 |
| ZBTB7B | ENSG00000198431 | TXNRD1 | GRIM-12, TR, TR1, TRXR1, TXNR |
| ZBTB7B | ENSG00000198520 | C1orf228 | NCRNA00082, p40 |
| ZBTB7B | ENSG00000198805 | PNP | NP, PRO1837, PUNP |
| ZBTB7B | ENSG00000198830 | HMGN2 | HMG17 |
| ZBTB7B | ENSG00000198841 | KTI12 | SBBI81, TOT4 |
| ZBTB7B | ENSG00000198858 | R3HDM4 | C19orf22 |
| ZBTB7B | ENSG00000198954 | KIAA1279 | KBP, KIAA1279, TTC20 |
| ZBTB7B | ENSG00000198976 | MIR429 | MIRN429, hsa-mir-429, mir-429 |
| ZBTB7B | ENSG00000198984 | MIR345 | MIRN345, hsa-mir-345, mir-345 |
| ZBTB7B | ENSG00000199032 | MIR425 | MIRN425, hsa-mir-425, mir-425 |
| ZBTB7B | ENSG00000199053 | MIR324 | MIRN324, hsa-mir-324, mir-324 |
| ZBTB7B | ENSG00000199394 | RNU6-600P | - |
| ZBTB7B | ENSG00000199490 | Y_RNA | - |
| ZBTB7B | ENSG00000199785 | SNORA52 | ACA52 |
| ZBTB7B | ENSG00000199787 | SNORA42 | - |
| ZBTB7B | ENSG00000200463 | SNORD118 | LCC, U8 |
| ZBTB7B | ENSG00000200646 | Y_RNA | - |
| ZBTB7B | ENSG00000201164 | RNU4-36P | - |
| ZBTB7B | ENSG00000201184 | RNU4-68P | - |
| ZBTB7B | ENSG00000201555 | Y_RNA | - |
| ZBTB7B | ENSG00000201675 | SNORD32A | RNU32, U32, U32A |
| ZBTB7B | ENSG00000203280 | CTA-221G9.12 | - |
| ZBTB7B | ENSG00000203499 | FAM83H-AS1 | onco-lncRNA-3 |
| ZBTB7B | ENSG00000203573 | Metazoa_SRP | - |
| ZBTB7B | ENSG00000204060 | FOXO6 | - |
| ZBTB7B | ENSG00000204070 | SYS1 | C20orf169, dJ453C12.4, dJ453C12.4.1 |
| ZBTB7B | ENSG00000204104 | TRAF3IP1 | IFT54, MIP-T3, MIPT3, SLSN9 |
| ZBTB7B | ENSG00000204209 | DAXX | BING2, DAP6, EAP1 |
| ZBTB7B | ENSG00000204237 | OXLD1 | C17orf90 |
| ZBTB7B | ENSG00000204301 | NOTCH4 | INT3 |
| ZBTB7B | ENSG00000204304 | PBX2 | G17, HOX12, PBX2MHC |
| ZBTB7B | ENSG00000204305 | AGER | RAGE, SCARJ1 |
| ZBTB7B | ENSG00000204308 | RNF5 | RING5, RMA1 |
| ZBTB7B | ENSG00000204310 | AGPAT1 | hsa-mir-6721, mir-6721 |
| ZBTB7B | ENSG00000204344 | STK19 | D6S60, D6S60E, G11, HLA-RP1, RP1 |
| ZBTB7B | ENSG00000204348 | DXO | DOM3L, DOM3Z, NG6, RAI1 |
| ZBTB7B | ENSG00000204351 | SKIV2L | 170A, DDX13, HLP, SKI2, SKI2W, SKIV2, SKIV2L1, THES2 |
| ZBTB7B | ENSG00000204356 | NELFE | MIRN1236, hsa-mir-1236 |
| ZBTB7B | ENSG00000204394 | VARS | G7A, VARS1, VARS2 |
| ZBTB7B | ENSG00000204396 | VWA7 | C6orf27, G7c, NG37 |
| ZBTB7B | ENSG00000204410 | MSH5 | G7, MUTSH5, NG23 |
| ZBTB7B | ENSG00000204420 | C6orf25 | G6b, G6b-B, NG31 |
| ZBTB7B | ENSG00000204421 | LY6G6C | C6orf24, G6c, NG24 |
| ZBTB7B | ENSG00000204427 | ABHD16A | BAT5, D6S82E, NG26, PP199 |
| ZBTB7B | ENSG00000204580 | DDR1 | - |
| ZBTB7B | ENSG00000204619 | PPP1R11 | CFAP255, HCG-V, HCGV, IPP3, TCTE5, TCTEX5 |
| ZBTB7B | ENSG00000204673 | AKT1S1 | Lobe, PRAS40 |
| ZBTB7B | ENSG00000204815 | TTC25 | CILD35 |
| ZBTB7B | ENSG00000204852 | TCTN1 | JBTS13, TECT1 |
| ZBTB7B | ENSG00000204930 | FAM221B | C9orf128 |
| ZBTB7B | ENSG00000204947 | ZNF425 | - |
| ZBTB7B | ENSG00000205084 | TMEM231 | ALYE870, JBTS20, MKS11, PRO1886 |
| ZBTB7B | ENSG00000205323 | SARNP | CIP29, HCC1, HSPC316, THO1 |
| ZBTB7B | ENSG00000205517 | RGL3 | - |
| ZBTB7B | ENSG00000205890 | RP11-473M20.5 | - |
| ZBTB7B | ENSG00000206145 | P2RX6P | - |
| ZBTB7B | ENSG00000206567 | AC022007.5 | - |
| ZBTB7B | ENSG00000206599 | RNU6-841P | - |
| ZBTB7B | ENSG00000207024 | Y_RNA | - |
| ZBTB7B | ENSG00000207574 | MIR661 | MIRN661, hsa-mir-661 |
| ZBTB7B | ENSG00000207605 | MIR191 | MIRN191, miR-191 |
| ZBTB7B | ENSG00000207607 | MIR200A | MIRN200A, mir-200a |
| ZBTB7B | ENSG00000207708 | MIR141 | MIRN141, mir-141 |
| ZBTB7B | ENSG00000207713 | MIR200C | MIRN200C, mir-200c |
| ZBTB7B | ENSG00000207730 | MIR200B | MIRN200B, mir-200b |
| ZBTB7B | ENSG00000207765 | AL132780.1 | - |
| ZBTB7B | ENSG00000207808 | MIR27A | MIR27, MIRN27A, mir-27a |
| ZBTB7B | ENSG00000207980 | MIR23A | MIRN23A, hsa-mir-23a, miRNA23A, mir-23a |
| ZBTB7B | ENSG00000208037 | MIR320A | MIRN320, MIRN320A, hsa-mir-320a, mir-320a |
| ZBTB7B | ENSG00000209645 | SNORD105 | U105 |
| ZBTB7B | ENSG00000212694 | LINC01089 | LIMT |
| ZBTB7B | ENSG00000213014 | VN2R17P | - |
| ZBTB7B | ENSG00000213024 | NUP62 | IBSN, SNDI, p62 |
| ZBTB7B | ENSG00000213085 | CFAP45 | CCDC19, NESG1 |
| ZBTB7B | ENSG00000213145 | CRIP1 | - |
| ZBTB7B | ENSG00000213339 | QTRT1 | FP3235, TGT, TGUT |
| ZBTB7B | ENSG00000213471 | TTLL13 | TTLL13 |
| ZBTB7B | ENSG00000213588 | ZBTB9 | ZNF919 |
| ZBTB7B | ENSG00000213600 | XXcos-LUCA16.1 | - |
| ZBTB7B | ENSG00000213654 | GPSM3 | AGS4, C6orf9, G18, G18.1a, G18.1b, G18.2, NG1 |
| ZBTB7B | ENSG00000213689 | TREX1 | - |
| ZBTB7B | ENSG00000213719 | CLIC1 | G6, NCC27 |
| ZBTB7B | ENSG00000213722 | DDAH2 | DDAH, DDAHII, G6a, HEL-S-277, NG30 |
| ZBTB7B | ENSG00000213753 | CENPBD1P1 | - |
| ZBTB7B | ENSG00000213859 | KCTD11 | C17orf36, KCASH1, REN, REN/KCTD11 |
| ZBTB7B | ENSG00000213920 | MDP1 | FN6PASE, MDP-1 |
| ZBTB7B | ENSG00000213983 | AP1G2 | G2AD |
| ZBTB7B | ENSG00000214063 | TSPAN4 | NAG-2, NAG2, TETRASPAN, TM4SF7, TSPAN-4 |
| ZBTB7B | ENSG00000214087 | ARL16 | - |
| ZBTB7B | ENSG00000214447 | FAM187A | CILD17, PR46b, SMH |
| ZBTB7B | ENSG00000214514 | KRT42P | - |
| ZBTB7B | ENSG00000214706 | IFRD2 | IFNRP, SKMc15, SM15 |
| ZBTB7B | ENSG00000215021 | PHB2 | BAP, BCAP37, Bap37, PNAS-141, REA, hBAP, p22 |
| ZBTB7B | ENSG00000215041 | NEURL4 | - |
| ZBTB7B | ENSG00000215183 | MSMP | PSMP |
| ZBTB7B | ENSG00000215692 | AC114730.8 | - |
| ZBTB7B | ENSG00000215908 | CROCCP2 | - |
| ZBTB7B | ENSG00000219355 | RPL31P52 | - |
| ZBTB7B | ENSG00000219435 | TEX40 | - |
| ZBTB7B | ENSG00000219481 | NBPF1 | AB13, AB14, AB23, AD2, NBG, NBPF |
| ZBTB7B | ENSG00000220205 | VAMP2 | - |
| ZBTB7B | ENSG00000221069 | AC000029.1 | - |
| ZBTB7B | ENSG00000221267 | MIR1236 | - |
| ZBTB7B | ENSG00000221400 | U3 | - |
| ZBTB7B | ENSG00000221829 | FANCG | FAG, XRCC9 |
| ZBTB7B | ENSG00000221838 | AP4M1 | CPSQ3, MU-4, MU-ARP2, SPG50 |
| ZBTB7B | ENSG00000221857 | CTD-2527I21.4 | - |
| ZBTB7B | ENSG00000221946 | FXYD7 | - |
| ZBTB7B | ENSG00000222011 | FAM185A | - |
| ZBTB7B | ENSG00000222028 | PSMB11 | BETA5T |
| ZBTB7B | ENSG00000222046 | DCDC2B | - |
| ZBTB7B | ENSG00000222152 | RNU6-1166P | - |
| ZBTB7B | ENSG00000223343 | RP13-131K19.2 | - |
| ZBTB7B | ENSG00000223382 | RP1-65J11.1 | - |
| ZBTB7B | ENSG00000223390 | RP11-91A18.4 | - |
| ZBTB7B | ENSG00000223823 | LINC01342 | - |
| ZBTB7B | ENSG00000224066 | RP4-622L5.7 | - |
| ZBTB7B | ENSG00000224077 | AP000936.4 | - |
| ZBTB7B | ENSG00000224265 | RP11-168L22.2 | - |
| ZBTB7B | ENSG00000224311 | RP11-40H20.4 | - |
| ZBTB7B | ENSG00000224647 | AC026954.6 | - |
| ZBTB7B | ENSG00000224758 | LINC01167 | - |
| ZBTB7B | ENSG00000224846 | RP1-90J20.8 | - |
| ZBTB7B | ENSG00000225265 | TAF1A-AS1 | - |
| ZBTB7B | ENSG00000225377 | NRSN2-AS1 | - |
| ZBTB7B | ENSG00000225889 | AC074289.1 | - |
| ZBTB7B | ENSG00000225931 | RP3-395M20.7 | - |
| ZBTB7B | ENSG00000225969 | ABHD11-AS1 | LINC00035, NCRNA00035, WBSCR26 |
| ZBTB7B | ENSG00000225981 | AC102953.4 | - |
| ZBTB7B | ENSG00000226328 | NUP50-AS1 | - |
| ZBTB7B | ENSG00000226330 | RP11-739N20.2 | - |
| ZBTB7B | ENSG00000227036 | LINC00511 | LCAL5, onco-lncRNA-12 |
| ZBTB7B | ENSG00000227589 | RP5-1092A11.5 | - |
| ZBTB7B | ENSG00000228037 | RP3-395M20.9 | - |
| ZBTB7B | ENSG00000228140 | RP3-467K16.4 | - |
| ZBTB7B | ENSG00000228146 | CASP16 | CASP16 |
| ZBTB7B | ENSG00000228201 | AL022341.3 | - |
| ZBTB7B | ENSG00000228300 | C19orf24 | - |
| ZBTB7B | ENSG00000228661 | AC090587.5 | - |
| ZBTB7B | ENSG00000228792 | RP11-354K1.2 | - |
| ZBTB7B | ENSG00000228843 | RP11-112J3.15 | - |
| ZBTB7B | ENSG00000229368 | AC090587.4 | - |
| ZBTB7B | ENSG00000229582 | RP11-423C15.3 | - |
| ZBTB7B | ENSG00000229766 | RP5-971N18.3 | - |
| ZBTB7B | ENSG00000229848 | RP13-766D20.2 | - |
| ZBTB7B | ENSG00000229947 | RP13-766D20.1 | - |
| ZBTB7B | ENSG00000229980 | TOB1-AS1 | - |
| ZBTB7B | ENSG00000230091 | TMEM254-AS1 | - |
| ZBTB7B | ENSG00000230454 | U73166.2 | - |
| ZBTB7B | ENSG00000230513 | THAP7-AS1 | - |
| ZBTB7B | ENSG00000230555 | RP11-517P14.2 | - |
| ZBTB7B | ENSG00000230615 | RP5-1198O20.4 | - |
| ZBTB7B | ENSG00000230955 | RP11-109P14.10 | - |
| ZBTB7B | ENSG00000231046 | RP11-428F8.2 | - |
| ZBTB7B | ENSG00000231057 | RP11-122M14.1 | - |
| ZBTB7B | ENSG00000231064 | RP11-263K19.4 | MIRN92B, hsa-mir-92b, mir-92b |
| ZBTB7B | ENSG00000231188 | RP11-34D15.2 | - |
| ZBTB7B | ENSG00000231233 | CCDC147-AS1 | - |
| ZBTB7B | ENSG00000231856 | RP11-327P2.5 | - |
| ZBTB7B | ENSG00000231925 | TAPBP | NGS17, TAPA, TPN, TPSN |
| ZBTB7B | ENSG00000231970 | RP11-452K12.7 | - |
| ZBTB7B | ENSG00000232093 | RP11-307C12.11 | - |
| ZBTB7B | ENSG00000232098 | CTD-2619J13.14 | - |
| ZBTB7B | ENSG00000232237 | ASCL5 | bHLHa47 |
| ZBTB7B | ENSG00000232306 | AC012485.2 | - |
| ZBTB7B | ENSG00000232352 | SEMA3B-AS1 | - |
| ZBTB7B | ENSG00000232434 | C9orf172 | - |
| ZBTB7B | ENSG00000232645 | LINC01431 | - |
| ZBTB7B | ENSG00000233246 | RP11-415J8.5 | - |
| ZBTB7B | ENSG00000233319 | PPIAP32 | - |
| ZBTB7B | ENSG00000233730 | RP4-666F24.3 | - |
| ZBTB7B | ENSG00000234181 | RP11-182N22.8 | - |
| ZBTB7B | ENSG00000234396 | RP11-181G12.4 | - |
| ZBTB7B | ENSG00000234432 | RP11-1275H24.1 | - |
| ZBTB7B | ENSG00000234684 | SDCBP2-AS1 | - |
| ZBTB7B | ENSG00000234936 | AC010883.5 | - |
| ZBTB7B | ENSG00000235058 | ZMYND10-AS1 | - |
| ZBTB7B | ENSG00000235084 | CHCHD2P6 | - |
| ZBTB7B | ENSG00000235162 | C12orf75 | AGD3, OCC-1, OCC1 |
| ZBTB7B | ENSG00000235236 | RP13-131K19.1 | - |
| ZBTB7B | ENSG00000235560 | AC002310.12 | - |
| ZBTB7B | ENSG00000235790 | RP11-73M7.6 | - |
| ZBTB7B | ENSG00000235823 | LINC00263 | - |
| ZBTB7B | ENSG00000236104 | ZBTB22 | BING1, ZBTB22A, ZNF297, ZNF297A, fru, fruitless |
| ZBTB7B | ENSG00000236263 | RP11-263K19.6 | - |
| ZBTB7B | ENSG00000236914 | RP11-1008C21.2 | - |
| ZBTB7B | ENSG00000237301 | RP4-680D5.2 | - |
| ZBTB7B | ENSG00000237441 | RGL2 | HKE1.5, KE1.5, RAB2L |
| ZBTB7B | ENSG00000237489 | LINC00959 | - |
| ZBTB7B | ENSG00000237775 | DDR1-AS1 | - |
| ZBTB7B | ENSG00000238164 | RP3-395M20.8 | - |
| ZBTB7B | ENSG00000238260 | RP11-46F15.2 | - |
| ZBTB7B | ENSG00000238279 | BX470102.3 | - |
| ZBTB7B | ENSG00000238758 | AL512791.1 | - |
| ZBTB7B | ENSG00000238795 | SCARNA12 | U89 |
| ZBTB7B | ENSG00000238923 | RNU7-1 | RNU7, U7.1 |
| ZBTB7B | ENSG00000238965 | RNA5SP351 | - |
| ZBTB7B | ENSG00000239160 | AL109947.1 | - |
| ZBTB7B | ENSG00000239617 | RP11-302B13.1 | - |
| ZBTB7B | ENSG00000239779 | WBP1 | WBP-1 |
| ZBTB7B | ENSG00000239930 | AP001625.4 | - |
| ZBTB7B | ENSG00000240230 | COX19 | - |
| ZBTB7B | ENSG00000240250 | RN7SL541P | - |
| ZBTB7B | ENSG00000240370 | RPL13P5 | RPL13-2, RPL13L, RPL13_4_1199, RRPL13L |
| ZBTB7B | ENSG00000240739 | RP11-69M1.1 | - |
| ZBTB7B | ENSG00000240877 | RN7SL521P | - |
| ZBTB7B | ENSG00000241146 | RPL7P41 | - |
| ZBTB7B | ENSG00000241258 | CRCP | CGRP-RCP, CGRPRCP, RCP, RCP9 |
| ZBTB7B | ENSG00000241420 | RN7SL505P | - |
| ZBTB7B | ENSG00000241709 | RN7SL265P | - |
| ZBTB7B | ENSG00000242852 | ZNF709 | - |
| ZBTB7B | ENSG00000243207 | PPAN-P2RY11 | - |
| ZBTB7B | ENSG00000243364 | EFNA4 | EFL4, EPLG4, LERK4 |
| ZBTB7B | ENSG00000243477 | NAT6 | FUS-2, FUS2 |
| ZBTB7B | ENSG00000243562 | RN7SL838P | - |
| ZBTB7B | ENSG00000243660 | ZNF487 | - |
| ZBTB7B | ENSG00000243976 | RN7SL523P | - |
| ZBTB7B | ENSG00000243989 | ACY1 | ACY-1, ACY1D, HEL-S-5 |
| ZBTB7B | ENSG00000244041 | LINC01011 | - |
| ZBTB7B | ENSG00000244065 | MARK2P17 | - |
| ZBTB7B | ENSG00000244187 | TMEM141 | - |
| ZBTB7B | ENSG00000245059 | RP11-303E16.7 | - |
| ZBTB7B | ENSG00000245156 | RP11-867G23.3 | - |
| ZBTB7B | ENSG00000245248 | USP2-AS1 | - |
| ZBTB7B | ENSG00000245532 | NEAT1 | LINC00084, NCRNA00084, TncRNA, VINC |
| ZBTB7B | ENSG00000245667 | RP5-940J5.8 | - |
| ZBTB7B | ENSG00000246548 | RP11-7F17.5 | - |
| ZBTB7B | ENSG00000246877 | DNM1P35 | - |
| ZBTB7B | ENSG00000248008 | DYNLL1-AS1 | DYNLL1-AS1, DYNLL1AS1 |
| ZBTB7B | ENSG00000248487 | ABHD14A | DORZ1 |
| ZBTB7B | ENSG00000248508 | SRP14-AS1 | - |
| ZBTB7B | ENSG00000248593 | DSTNP2 | - |
| ZBTB7B | ENSG00000249319 | AC068533.7 | - |
| ZBTB7B | ENSG00000249471 | ZNF324B | - |
| ZBTB7B | ENSG00000251161 | RP11-540O11.1 | - |
| ZBTB7B | ENSG00000251169 | AC005355.2 | - |
| ZBTB7B | ENSG00000251246 | RP11-540D14.8 | - |
| ZBTB7B | ENSG00000251503 | APITD1-CORT | - |
| ZBTB7B | ENSG00000252211 | RNA5SP473 | - |
| ZBTB7B | ENSG00000252334 | RNU6-1337P | - |
| ZBTB7B | ENSG00000252743 | RNU6-850P | - |
| ZBTB7B | ENSG00000253408 | RP11-231D20.2 | - |
| ZBTB7B | ENSG00000253720 | RP11-473O4.3 | - |
| ZBTB7B | ENSG00000254389 | RHPN1-AS1 | C8orf51 |
| ZBTB7B | ENSG00000254445 | HSPB2-C11orf52 | - |
| ZBTB7B | ENSG00000254452 | RP11-867G23.4 | - |
| ZBTB7B | ENSG00000254461 | RP11-755F10.3 | - |
| ZBTB7B | ENSG00000254501 | AP003068.9 | - |
| ZBTB7B | ENSG00000254505 | CHMP4A | C14orf123, CHMP4, CHMP4B, HSPC134, SHAX2, SNF7, SNF7-1, VPS32-1, VPS32A |
| ZBTB7B | ENSG00000254545 | RP11-84A19.3 | - |
| ZBTB7B | ENSG00000254548 | RP11-429J17.5 | - |
| ZBTB7B | ENSG00000254574 | RP11-429J17.4 | - |
| ZBTB7B | ENSG00000254578 | CTD-2517M22.16 | - |
| ZBTB7B | ENSG00000254692 | TM9SF1 | - |
| ZBTB7B | ENSG00000254740 | RP11-334E6.3 | - |
| ZBTB7B | ENSG00000254756 | RP11-867G23.12 | - |
| ZBTB7B | ENSG00000254762 | RP11-867G23.2 | - |
| ZBTB7B | ENSG00000254806 | SYS1-DBNDD2 | C20orf169-DBNDD2 |
| ZBTB7B | ENSG00000254827 | SLC22A18AS | BWR1B, BWSCR1B, ORCTL2S, SLC22A1LS, p27-BWR1B |
| ZBTB7B | ENSG00000254855 | RP11-867G23.1 | - |
| ZBTB7B | ENSG00000255108 | AP006621.8 | - |
| ZBTB7B | ENSG00000255119 | RP11-655M14.12 | - |
| ZBTB7B | ENSG00000255152 | MSH5-SAPCD1 | MSH5-C6orf26 |
| ZBTB7B | ENSG00000255173 | AP003068.12 | - |
| ZBTB7B | ENSG00000255182 | CTD-2517M22.14 | - |
| ZBTB7B | ENSG00000255468 | RP11-867G23.8 | - |
| ZBTB7B | ENSG00000255572 | RP11-273B20.3 | - |
| ZBTB7B | ENSG00000255737 | AGAP2-AS1 | PUNISHER |
| ZBTB7B | ENSG00000255966 | RP5-940J5.3 | - |
| ZBTB7B | ENSG00000256053 | APOPT1 | APOP, APOP1, C14orf153 |
| ZBTB7B | ENSG00000256061 | DYX1C1 | CILD25, DNAAF4, DYX1, DYXC1, EKN1, RD |
| ZBTB7B | ENSG00000256116 | RP11-783K16.14 | - |
| ZBTB7B | ENSG00000256349 | CTD-3074O7.11 | BBS2L2 |
| ZBTB7B | ENSG00000256433 | RP1-102E24.8 | - |
| ZBTB7B | ENSG00000256500 | RP11-73M18.2 | - |
| ZBTB7B | ENSG00000256940 | RP11-783K16.5 | - |
| ZBTB7B | ENSG00000257069 | RP11-783K16.10 | - |
| ZBTB7B | ENSG00000257084 | U47924.27 | - |
| ZBTB7B | ENSG00000257086 | RP11-783K16.13 | - |
| ZBTB7B | ENSG00000257218 | GATC | 15E1.2 |
| ZBTB7B | ENSG00000257270 | RP11-521B24.5 | - |
| ZBTB7B | ENSG00000257341 | CRIP1 | CRHP, CRIP, CRP-1, CRP1 |
| ZBTB7B | ENSG00000257384 | RP11-644F5.12 | - |
| ZBTB7B | ENSG00000257605 | RP11-680A11.5 | - |
| ZBTB7B | ENSG00000257642 | RP11-474B16.1 | - |
| ZBTB7B | ENSG00000257921 | RP11-571M6.15 | - |
| ZBTB7B | ENSG00000257949 | TEN1 | - |
| ZBTB7B | ENSG00000258045 | Metazoa_SRP | - |
| ZBTB7B | ENSG00000258056 | RP11-644F5.11 | - |
| ZBTB7B | ENSG00000258311 | RP11-644F5.10 | - |
| ZBTB7B | ENSG00000258325 | RP4-816N1.6 | - |
| ZBTB7B | ENSG00000258727 | RP11-66N24.3 | - |
| ZBTB7B | ENSG00000258740 | RP11-293M10.1 | - |
| ZBTB7B | ENSG00000258820 | RP11-293M10.2 | - |
| ZBTB7B | ENSG00000258914 | CTD-2134A5.3 | - |
| ZBTB7B | ENSG00000259146 | RP1-261D10.2 | - |
| ZBTB7B | ENSG00000259431 | THTPA | THTP, THTPASE |
| ZBTB7B | ENSG00000259448 | RP11-16E12.1 | - |
| ZBTB7B | ENSG00000259605 | AC074212.5 | - |
| ZBTB7B | ENSG00000259687 | LINC01220 | - |
| ZBTB7B | ENSG00000259700 | RP11-485O10.3 | - |
| ZBTB7B | ENSG00000259840 | LA16c-380A1.1 | - |
| ZBTB7B | ENSG00000259920 | RP11-2E11.5 | - |
| ZBTB7B | ENSG00000260007 | RP11-315D16.2 | - |
| ZBTB7B | ENSG00000260038 | RP11-407G23.4 | - |
| ZBTB7B | ENSG00000260259 | RP11-368I7.4 | - |
| ZBTB7B | ENSG00000260285 | RP11-600F24.7 | - |
| ZBTB7B | ENSG00000260288 | RP11-24M17.4 | - |
| ZBTB7B | ENSG00000260394 | LA16c-313D11.9 | - |
| ZBTB7B | ENSG00000260490 | RP11-265N6.3 | - |
| ZBTB7B | ENSG00000260494 | AC002310.10 | - |
| ZBTB7B | ENSG00000260563 | RP13-516M14.1 | - |
| ZBTB7B | ENSG00000260643 | RP11-303E16.8 | - |
| ZBTB7B | ENSG00000260669 | AL136419.6 | - |
| ZBTB7B | ENSG00000260869 | AC002310.13 | - |
| ZBTB7B | ENSG00000261147 | RP11-697E2.6 | - |
| ZBTB7B | ENSG00000261221 | ZNF865 | - |
| ZBTB7B | ENSG00000261408 | TEN1-CDK3 | C17orf106 |
| ZBTB7B | ENSG00000261459 | ZNF747 | - |
| ZBTB7B | ENSG00000261526 | CTB-31O20.2 | - |
| ZBTB7B | ENSG00000261659 | LA16c-313D11.12 | - |
| ZBTB7B | ENSG00000261702 | RP11-282M16.1 | - |
| ZBTB7B | ENSG00000261771 | DYX1C1-CCPG1 | - |
| ZBTB7B | ENSG00000261790 | AC005606.14 | - |
| ZBTB7B | ENSG00000261840 | RP11-146F11.1 | - |
| ZBTB7B | ENSG00000261949 | GFY | Goofy |
| ZBTB7B | ENSG00000262141 | CTC-479C5.11 | - |
| ZBTB7B | ENSG00000262302 | RP1-4G17.5 | - |
| ZBTB7B | ENSG00000262528 | LA16c-349E10.1 | - |
| ZBTB7B | ENSG00000262533 | RP11-667K14.4 | - |
| ZBTB7B | ENSG00000262583 | RP11-77K12.5 | - |
| ZBTB7B | ENSG00000262664 | OVCA2 | - |
| ZBTB7B | ENSG00000263011 | RP11-473M20.11 | - |
| ZBTB7B | ENSG00000263072 | ZNF213-AS1 | - |
| ZBTB7B | ENSG00000263171 | RP11-542C16.1 | - |
| ZBTB7B | ENSG00000263214 | ZNF205-AS1 | - |
| ZBTB7B | ENSG00000263280 | LA16c-325D7.2 | - |
| ZBTB7B | ENSG00000263325 | LA16c-325D7.1 | - |
| ZBTB7B | ENSG00000263421 | MIR4687 | - |
| ZBTB7B | ENSG00000263506 | MIR5193 | - |
| ZBTB7B | ENSG00000263519 | AL157871.1 | - |
| ZBTB7B | ENSG00000263620 | RP11-599B13.6 | SYB2, VAMP-2 |
| ZBTB7B | ENSG00000263800 | MIR5684 | - |
| ZBTB7B | ENSG00000263853 | AC139530.1 | - |
| ZBTB7B | ENSG00000264078 | RP11-73M7.9 | - |
| ZBTB7B | ENSG00000264085 | MIR5004 | - |
| ZBTB7B | ENSG00000264357 | MIR4648 | - |
| ZBTB7B | ENSG00000264594 | MIR4640 | - |
| ZBTB7B | ENSG00000264624 | MIR3615 | - |
| ZBTB7B | ENSG00000264749 | AC011313.1 | - |
| ZBTB7B | ENSG00000264779 | AC107871.1 | - |
| ZBTB7B | ENSG00000265039 | AC107016.1 | - |
| ZBTB7B | ENSG00000265100 | RP11-147L13.2 | - |
| ZBTB7B | ENSG00000265386 | RN7SL219P | - |
| ZBTB7B | ENSG00000265388 | RN7SL391P | - |
| ZBTB7B | ENSG00000265660 | MIR4664 | - |
| ZBTB7B | ENSG00000265692 | RP13-516M14.4 | - |
| ZBTB7B | ENSG00000265700 | MIR4647 | - |
| ZBTB7B | ENSG00000266036 | MIR3615 | - |
| ZBTB7B | ENSG00000266070 | MIR3960 | - |
| ZBTB7B | ENSG00000266077 | AC139149.1 | - |
| ZBTB7B | ENSG00000266458 | hsa-mir-4259 | - |
| ZBTB7B | ENSG00000266465 | AC025165.1 | - |
| ZBTB7B | ENSG00000266559 | MIR4530 | - |
| ZBTB7B | ENSG00000266640 | MIR4754 | mir-4754 |
| ZBTB7B | ENSG00000266824 | RP11-599B13.7 | - |
| ZBTB7B | ENSG00000266907 | AC006116.22 | - |
| ZBTB7B | ENSG00000266964 | FXYD1 | PLM |
| ZBTB7B | ENSG00000266995 | RP11-703I16.3 | - |
| ZBTB7B | ENSG00000267007 | CTB-31O20.3 | - |
| ZBTB7B | ENSG00000267062 | CTD-2659N19.10 | - |
| ZBTB7B | ENSG00000267128 | RNF157-AS1 | - |
| ZBTB7B | ENSG00000267141 | CTB-31O20.8 | - |
| ZBTB7B | ENSG00000267159 | AC005391.2 | - |
| ZBTB7B | ENSG00000267169 | CTB-55O6.12 | - |
| ZBTB7B | ENSG00000267212 | CTD-2659N19.9 | - |
| ZBTB7B | ENSG00000267219 | AC010504.2 | - |
| ZBTB7B | ENSG00000267232 | CTB-31O20.9 | - |
| ZBTB7B | ENSG00000267253 | RP11-209M4.1 | - |
| ZBTB7B | ENSG00000267262 | CTC-232P5.3 | - |
| ZBTB7B | ENSG00000267277 | CTD-2342J14.6 | - |
| ZBTB7B | ENSG00000267298 | AC006116.19 | - |
| ZBTB7B | ENSG00000267299 | CTB-66B24.1 | - |
| ZBTB7B | ENSG00000267334 | CTD-2534I21.8 | - |
| ZBTB7B | ENSG00000267395 | AC074212.6 | - |
| ZBTB7B | ENSG00000267493 | CIRBP-AS1 | - |
| ZBTB7B | ENSG00000267519 | CTD-3252C9.4 | - |
| ZBTB7B | ENSG00000267549 | AC006116.17 | - |
| ZBTB7B | ENSG00000267589 | CTC-232P5.4 | - |
| ZBTB7B | ENSG00000267615 | RP11-552F3.13 | - |
| ZBTB7B | ENSG00000267695 | RP11-1030E3.1 | - |
| ZBTB7B | ENSG00000267737 | AC061992.2 | - |
| ZBTB7B | ENSG00000267811 | RP11-727F15.11 | - |
| ZBTB7B | ENSG00000267858 | MZF1-AS1 | - |
| ZBTB7B | ENSG00000267896 | AC018766.4 | - |
| ZBTB7B | ENSG00000268049 | CTD-2619J13.9 | - |
| ZBTB7B | ENSG00000268230 | CTD-2619J13.8 | - |
| ZBTB7B | ENSG00000268307 | CTD-2619J13.13 | - |
| ZBTB7B | ENSG00000268366 | CTC-492K19.4 | - |
| ZBTB7B | ENSG00000268465 | CTC-273B12.7 | - |
| ZBTB7B | ENSG00000268530 | CTC-273B12.5 | - |
| ZBTB7B | ENSG00000268543 | CTD-2619J13.16 | - |
| ZBTB7B | ENSG00000268583 | CTC-453G23.8 | - |
| ZBTB7B | ENSG00000268784 | MGC2752 | - |
| ZBTB7B | ENSG00000269032 | AC016629.7 | - |
| ZBTB7B | ENSG00000269038 | AP001462.6 | - |
| ZBTB7B | ENSG00000269054 | CTD-2619J13.3 | - |
| ZBTB7B | ENSG00000269058 | CALR3 | CMH19, CRT2, CT93 |
| ZBTB7B | ENSG00000269095 | AC010646.3 | - |
| ZBTB7B | ENSG00000269106 | CTD-2619J13.23 | - |
| ZBTB7B | ENSG00000269176 | RP11-727F15.12 | - |
| ZBTB7B | ENSG00000269194 | AC006942.4 | - |
| ZBTB7B | ENSG00000269307 | CTD-2278I10.6 | - |
| ZBTB7B | ENSG00000269352 | PTOV1-AS2 | - |
| ZBTB7B | ENSG00000269399 | CTD-3222D19.12 | - |
| ZBTB7B | ENSG00000269559 | RP11-629B11.5 | - |
| ZBTB7B | ENSG00000269751 | CTC-273B12.8 | - |
| ZBTB7B | ENSG00000269755 | CTD-3105H18.18 | - |
| ZBTB7B | ENSG00000269855 | RNF225 | - |
| ZBTB7B | ENSG00000269926 | RP11-442H21.2 | - |
| ZBTB7B | ENSG00000269968 | RP5-940J5.9 | - |
| ZBTB7B | ENSG00000269971 | RP3-426I6.5 | - |
| ZBTB7B | ENSG00000270598 | RP11-396C23.3 | - |
| ZBTB7B | ENSG00000271065 | RP11-607P23.1 | - |
| ZBTB7B | ENSG00000271236 | SUMO2P15 | - |
| ZBTB7B | ENSG00000271732 | RP5-1182A14.5 | - |
| ZBTB7B | ENSG00000271748 | MIR92B | - |
| ZBTB7B | ENSG00000271969 | U47924.29 | - |
| ZBTB7B | ENSG00000271989 | RP4-736L20.3 | - |
| ZBTB7B | ENSG00000272104 | XXcos-LUCA11.5 | - |
| ZBTB7B | ENSG00000272141 | RP11-465B22.8 | - |
| ZBTB7B | ENSG00000272173 | RNU7-1 | - |
| ZBTB7B | ENSG00000272434 | RP13-131K19.6 | - |
| ZBTB7B | ENSG00000272449 | RP3-395M20.12 | - |
| ZBTB7B | ENSG00000272663 | RP11-191L17.1 | - |
| ZBTB7B | ENSG00000272701 | RP11-2E11.10 | - |
| ZBTB7B | ENSG00000272762 | RP11-155D18.12 | - |
| ZBTB7B | ENSG00000272849 | RP11-347I19.8 | - |
| ZBTB7B | ENSG00000272953 | RP11-1275H24.2 | - |
| ZBTB7B | ENSG00000273025 | CELF6 | BRUNOL6 |
| ZBTB7B | ENSG00000273084 | RP11-1275H24.3 | - |
| ZBTB7B | ENSG00000273145 | CITF22-92A6.1 | - |
| ZBTB7B | ENSG00000273179 | RP11-20I20.4 | - |
| ZBTB7B | ENSG00000273211 | RP13-131K19.7 | - |
| ZBTB7B | ENSG00000273230 | RP11-1246C19.1 | - |
| ZBTB7B | ENSG00000273333 | XXbac-BPG300A18.13 | - |
| ZBTB7B | ENSG00000273335 | RP11-61L19.2 | - |
| ZBTB7B | ENSG00000273523 | RP11-248G5.9 | - |
| ZBTB7B | ENSG00000273559 | CWC25 | CCDC49 |
| ZBTB7B | ENSG00000274049 | INO80B-WBP1 | - |
| ZBTB7B | ENSG00000274054 | MIR4727 | mir-4727 |
| ZBTB7B | ENSG00000274259 | XXbac-BPG294E21.9 | - |
| ZBTB7B | ENSG00000274292 | RP11-347I19.7 | - |
| ZBTB7B | ENSG00000274388 | AC141002.1 | - |
| ZBTB7B | ENSG00000274596 | MIR6872 | - |
| ZBTB7B | ENSG00000274856 | MIR6748 | - |
| ZBTB7B | ENSG00000274897 | PANO | PANO |
| ZBTB7B | ENSG00000274904 | CTD-2515O10.5 | - |
| ZBTB7B | ENSG00000274937 | CTD-2311M21.4 | - |
| ZBTB7B | ENSG00000275175 | CTD-2313J17.7 | - |
| ZBTB7B | ENSG00000275198 | RP11-471B22.3 | - |
| ZBTB7B | ENSG00000275200 | AP001885.1 | - |
| ZBTB7B | ENSG00000275527 | CTD-3154N5.2 | - |
| ZBTB7B | ENSG00000275635 | U7 | - |
| ZBTB7B | ENSG00000275703 | U47924.32 | - |
| ZBTB7B | ENSG00000275726 | MIR6088 | hsa-mir-6088 |
| ZBTB7B | ENSG00000275734 | RP11-368I7.6 | - |
| ZBTB7B | ENSG00000275924 | MIR6807 | hsa-mir-6807 |
| ZBTB7B | ENSG00000276408 | RP11-490B18.5 | - |
| ZBTB7B | ENSG00000276511 | MIR7703 | - |
| ZBTB7B | ENSG00000276523 | RP11-490B18.6 | - |
| ZBTB7B | ENSG00000276584 | MIR6737 | hsa-mir-6737 |
| ZBTB7B | ENSG00000276663 | RP11-407G23.7 | - |
| ZBTB7B | ENSG00000276698 | RP11-468E2.10 | - |
| ZBTB7B | ENSG00000276797 | MIR24-2 | - |
| ZBTB7B | ENSG00000277235 | RP4-550H1.7 | - |
| ZBTB7B | ENSG00000277249 | MIR6784 | hsa-mir-6784 |
| ZBTB7B | ENSG00000277264 | MIR6833 | hsa-mir-6833 |
| ZBTB7B | ENSG00000277269 | MESTIT1_1 | - |
| ZBTB7B | ENSG00000277363 | SRCIN1 | P140, SNIP |
| ZBTB7B | ENSG00000277453 | CTC-492K19.7 | - |
| ZBTB7B | ENSG00000277510 | Metazoa_SRP | - |
| ZBTB7B | ENSG00000277525 | U2 | - |
| ZBTB7B | ENSG00000277547 | MIR6751 | - |
| ZBTB7B | ENSG00000277599 | NEAT1_3 | - |
| ZBTB7B | ENSG00000277784 | MIR6786 | hsa-mir-6786 |
| ZBTB7B | ENSG00000278050 | NEAT1_2 | - |
| ZBTB7B | ENSG00000278055 | MESTIT1 | - |
| ZBTB7B | ENSG00000278144 | NEAT1_1 | - |
| ZBTB7B | ENSG00000278185 | RP11-153F5.7 | - |
| ZBTB7B | ENSG00000278200 | RP13-766D20.4 | - |
| ZBTB7B | ENSG00000278663 | MIR6887 | - |
| ZBTB7B | ENSG00000278784 | RP11-468E2.11 | - |
| ZBTB7B | ENSG00000278922 | AC002310.14 | - |
| ZBTB7B | ENSG00000278952 | RP11-399J13.2 | - |
| ZBTB7B | ENSG00000278954 | RP13-415G19.2 | - |
| ZBTB7B | ENSG00000278995 | Z69720.3 | - |
| ZBTB7B | ENSG00000279199 | RP11-749I16.3 | - |
| ZBTB7B | ENSG00000279255 | LA16c-380A1.2 | - |
| ZBTB7B | ENSG00000279441 | LA16c-313D11.13 | - |
| ZBTB7B | ENSG00000279487 | AC234582.1 | - |
| ZBTB7B | ENSG00000279619 | CTD-2527I21.5 | - |
| ZBTB7B | ENSG00000279742 | RP11-700A24.1 | - |
| ZBTB7B | ENSG00000279873 | LINC01126 | - |
| ZBTB7B | ENSG00000279986 | AC234582.2 | - |
| ZBTB7B | ENSG00000280035 | RP11-10J21.2 | - |
| ZBTB7B | ENSG00000280420 | AC005355.3 | - |
| ZBTB7B | ENSG00000280424 | CITF22-92A6.2 | - |
