# Supplementary figures and images for: ThPOK inhibits the immune escape of gastric cancer cells by inducing STPG1 to inactivate the ERK pathway
Source: BMC Immunol. 2022 Apr 4;23:16. doi: 10.1186/s12865-022-00485-5 (PMC8981657; doi:10.1186/s12865-022-00485-5)

1A

Thpok

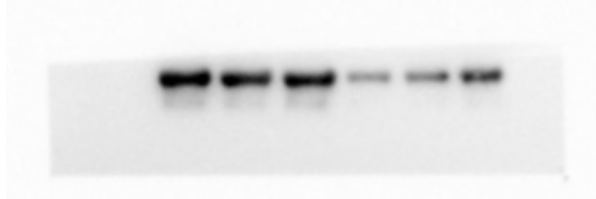

GAPDH

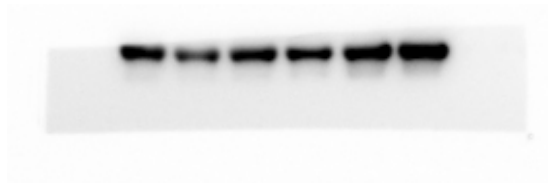

1B

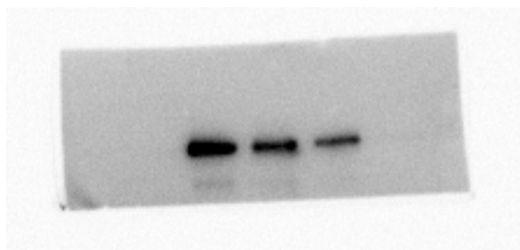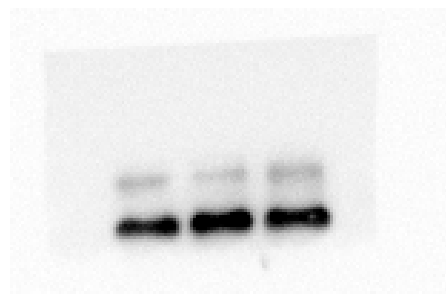

3C

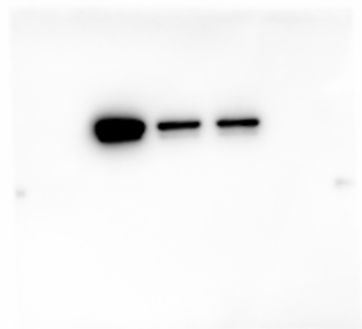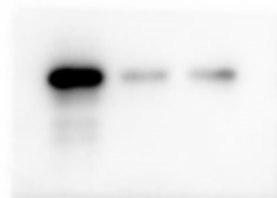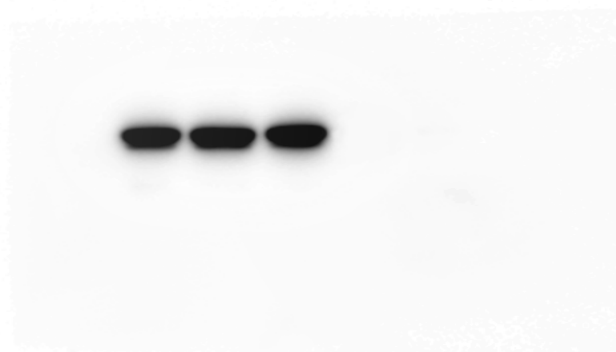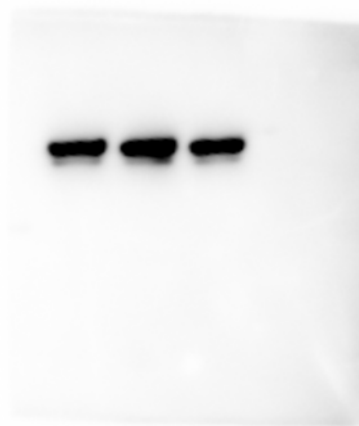

3D

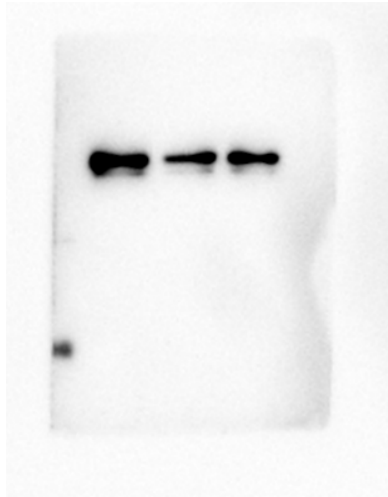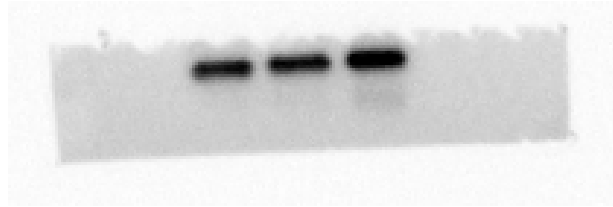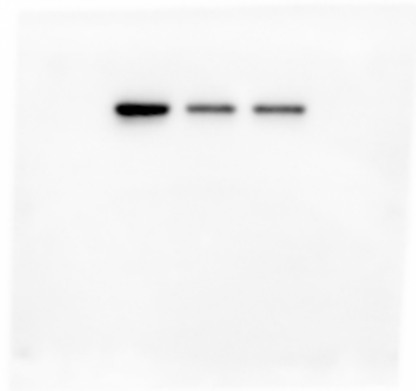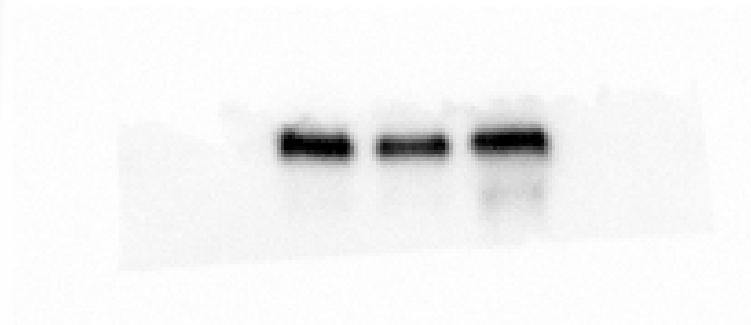

5A

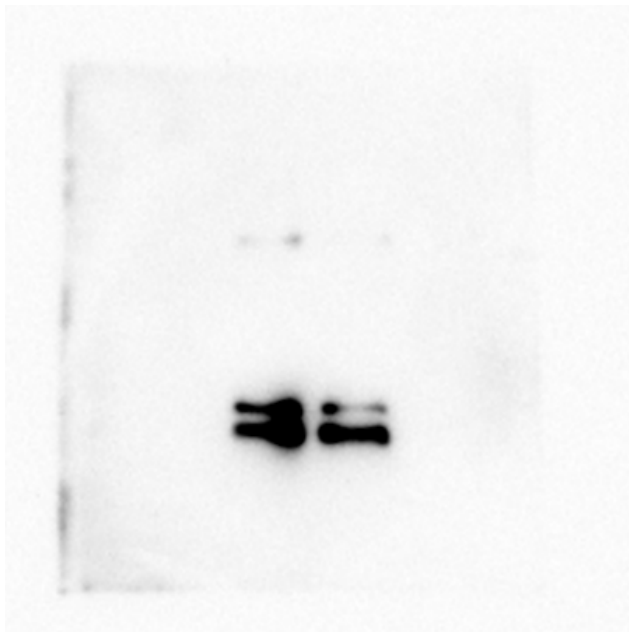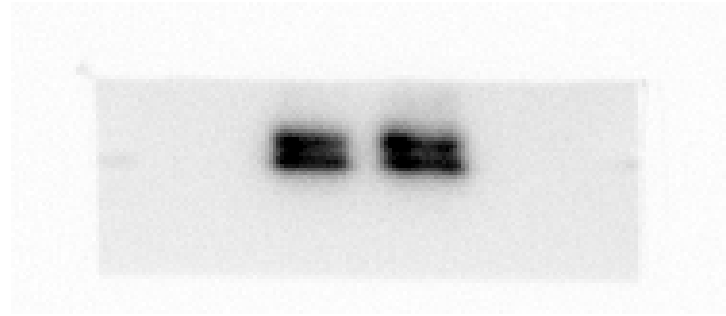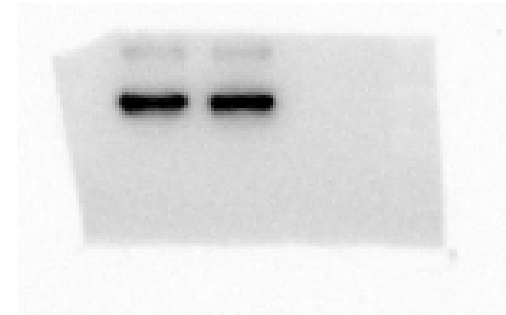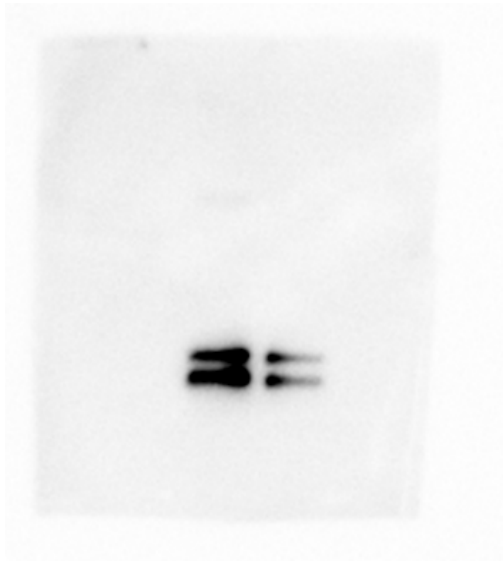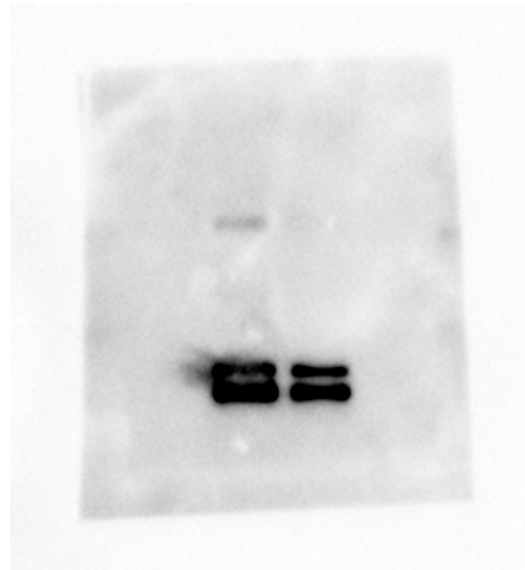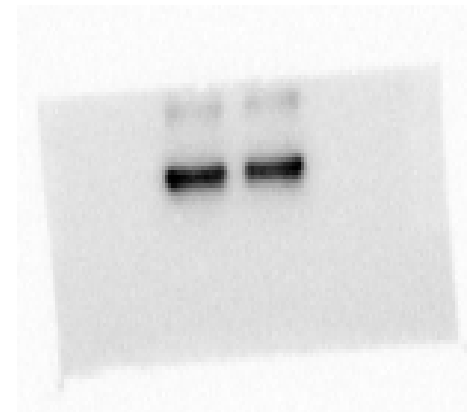

5C

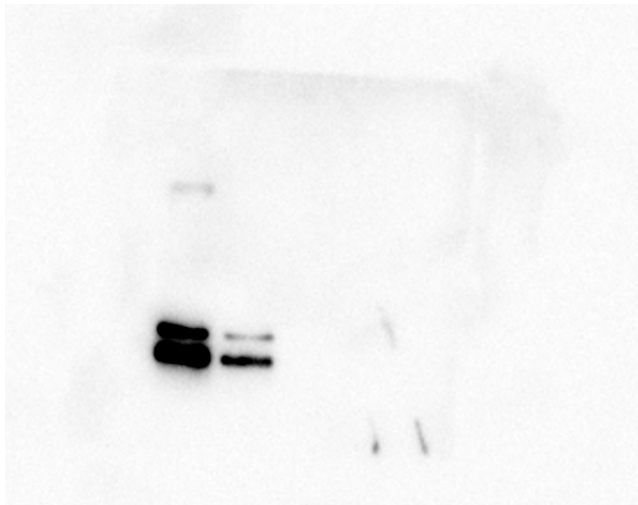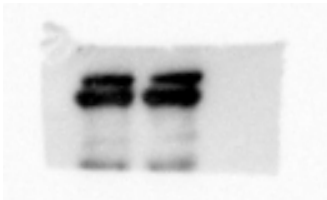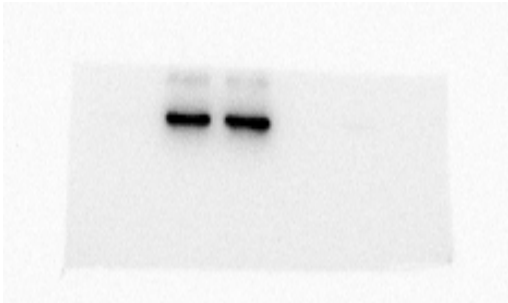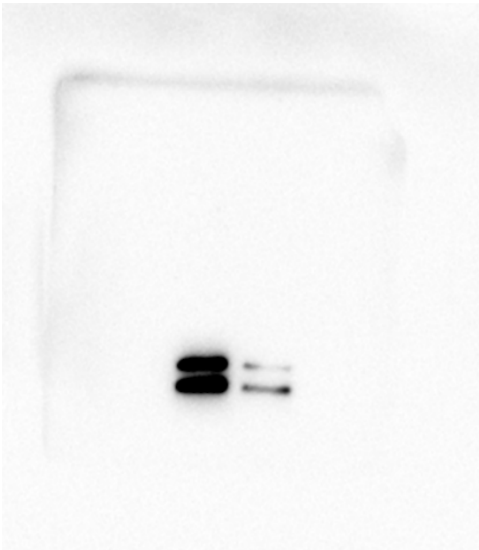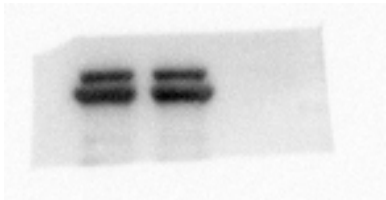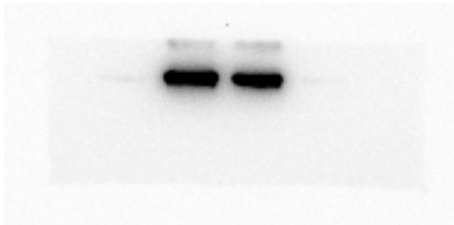

5E

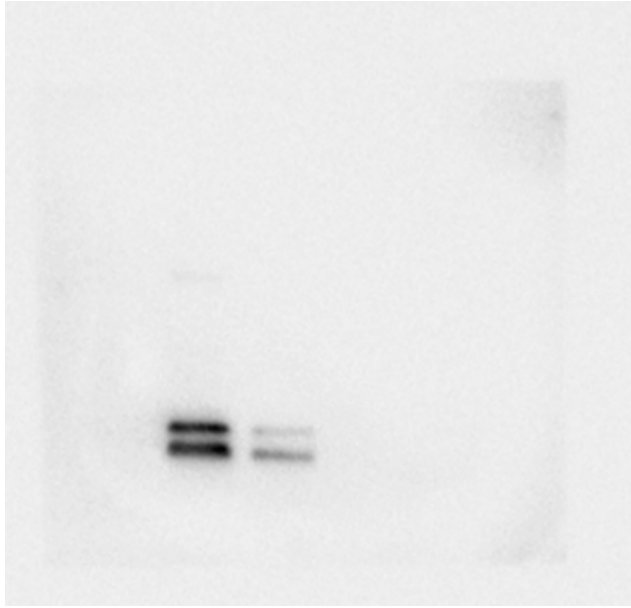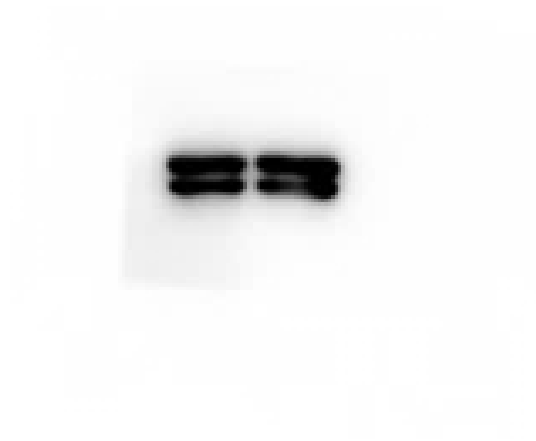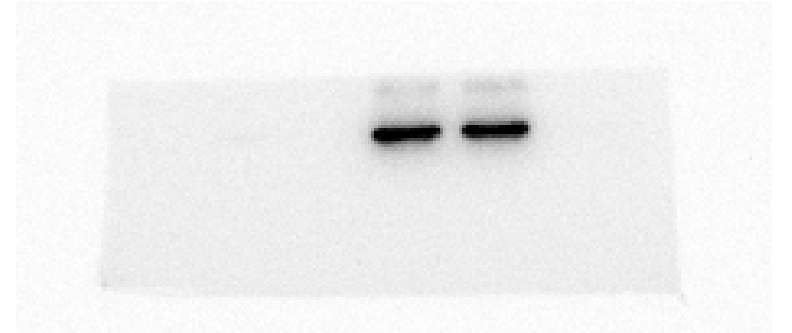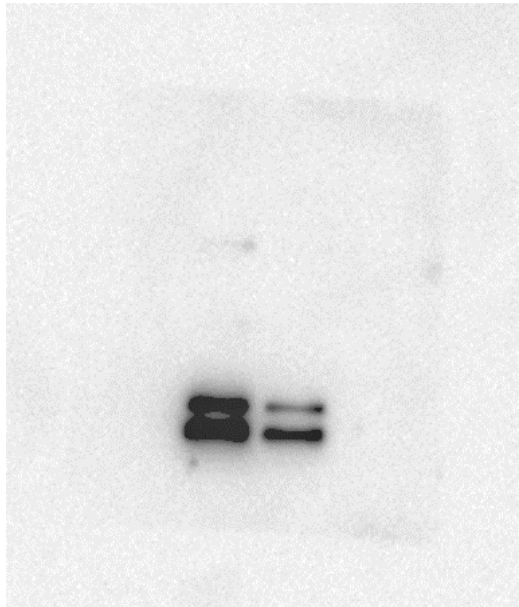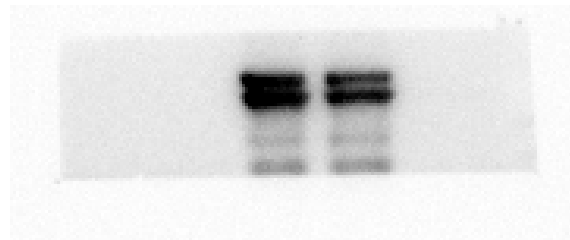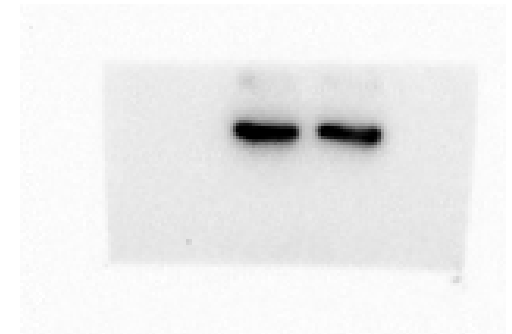

Supplement: Supplementary file 2 — Additional file 2. Original western blot images. [file 12865_2022_485_MOESM2_ESM.pdf]
